# Polyamide membranes with nanoscale ordered structures for fast permeation and highly selective ion-ion separation

**DOI:** 10.1038/s41467-023-36848-8

**Published:** 2023-02-27

**Authors:** Changwei Zhao, Yanjun Zhang, Yuewen Jia, Bojun Li, Wenjing Tang, Chuning Shang, Rui Mo, Pei Li, Shaomin Liu, Sui Zhang

**Affiliations:** 1 College of Resources and Environmental Sciences, China Agricultural University, Beijing, 100193 China; 2 Department of Chemical and Biomolecular Engineering, National University of Singapore, 4 Engineering Drive 4, 117585 Singapore, Singapore; 3 State Key Laboratory of Water Environment Simulation, School of Environment, Beijing Normal University, Beijing, 100875 China; 4 College of Materials Science and Engineering, College of Chemical Engineering, Beijing University of Chemical Technology, 100029 Beijing, China

**Keywords:** Chemical engineering, Structural properties, Pollution remediation

## Abstract

Fast permeation and effective solute-solute separation provide the opportunities for sustainable water treatment, but they are hindered by ineffective membranes. We present here the construction of a nanofiltration membrane with fast permeation, high rejection, and precise Cl^-^/SO_4_^2-^ separation by spatial and temporal control of interfacial polymerization via graphitic carbon nitride (g-C_3_N_4_). The g-C_3_N_4_ nanosheet binds preferentially with piperazine and tiles the water-hexane interface as revealed by molecular dynamics studies, thus lowering the diffusion rate of PIP by one order of magnitude and restricting its diffusion pathways towards the hexane phase. As a result, membranes with nanoscale ordered hollow structure are created. Transport mechanism across the structure is clarified using computational fluid dynamics simulation. Increased surface area, lower thickness, and a hollow ordered structure are identified as the key contributors to the water permeance of 105 L m^2^·h^−1^·bar^−1^ with a Na_2_SO_4_ rejection of 99.4% and a Cl^-^/SO_4_^2-^ selectivity of 130, which is superior to state-of-the-art NF membranes. Our approach for tuning the membrane microstructure enables the development of ultra-permeability and excellent selectivity for ion-ion separation, water purification, desalination, and organics removal.

## Abstract (web summary)

Membranes with precise ion-ion separation are critical for sustainable water treatment. Here, authors demonstrated controlled construction of a nanofiltration membrane with fast permeation and high Cl^-^/SO_4_^2-^ selectivity by simultaneous spatial and temporal control of interfacial polymerization.

## Introduction

Nanofiltration (NF) is a cost-effective and environmentally friendly separation method used in municipal wastewater treatment, drinking water purification, and pharmaceutical purification^1–5^. Compared to other technologies, NF consumes lower energy, takes up less space, and can effectively separate ions. A durable membrane with desirable permeability and selectivity is essential for establishing the ideal NF process. A high membrane permeability allows for operation at lower pressures, further reducing the energy cost. Another important criterion is selectivity. Many studies to date have emphasized the selectivity of water over solutes in NF applications^6^. In recent years, solute-solute separation has attracted increasing attention as a value-added process to water treatment, which may enable many possibilities, such as the energy-effective removal of a specific solute from the water stream and isolation and concentration of a valuable component^7^. One typical example is the separation of chloride from sulfate in the chloralkali process. Cl^−^/SO_4_^2−^ separation is beneficial for recovering valuable salt resources and obtaining clean freshwater^8–13^.

NF membranes offer a wide range of applications due to their diversified pore size and charge characteristics^14–17^. Their molecular weight cut-off (MWCO) typically ranges between 200 and 1000 Da, and membrane surfaces can be positive, negative, neutral, or zwitterionic. The design of NF membranes with appropriate microstructure, thickness, pore size, and surface charge should be tailored to suit each specific application. Most commercial NF membranes are now manufactured by the interfacial polymerization (IP) of piperazine (PIP) in the aqueous phase and trimesoyl chloride (TMC) in the organic phase^18–20^. Polyamide-based thin film composite membranes remain the gold standard in both the market and academic research^21,22^. The quick and less regulated reaction, however, makes it challenging to tune the structure of the resulting polyamide layer^23,24^. As a result, NF membranes are subjected to an inherent trade-off between flux and selectivity.

Many recent efforts have been made to enhance the permeability by experimenting with various reaction conditions, such as modifying the compositions of the aqueous and organic phases, incorporating nano-fillers, and employing layer-by-layer and 3D printing technologies^25–31^. These strategies seek to manage the IP process by either slowing down the reaction or confining the reaction in a limited space^6^. As a result, the impact on membrane structure and separation performances is limited. Furthermore, most studies do not address solute–solute selectivity.

Herein, a graphitic carbon nitride (g-C_3_N_4_) nanosheet was adopted during IP to form an ordered nanoscale hollow structure of a thin-film composite membrane. Molecular dynamics (MD) investigations revealed that g-C_3_N_4_ plays a critical role in temporally controlling the IP process by slowing PIP diffusion and spatially restricting the reaction via tiling at the oil–water interface. PIP diffusivity was reduced by one order of magnitude in experiments, and an NF membrane with a nanoscale-ordered hollow cone structure was created, the size and distribution of which were depending on g-C_3_N_4_ concentration. The impact of such a structure on the transport mechanism was investigated further. The membrane exhibited high permeance and outstanding Cl^−^/SO_4_^2^^−^ selectivity.

Notably, g-C_3_N_4_ has been used in prior studies as nanofillers^32^ or interlayer^33^ during IP. In this work, we prepared g-C_3_N_4_ suspensions with high concentrations that induce spatial and temporal modulation to get a nanoscale-ordered morphology and extraordinary separation capacities, and the role of g-C_3_N_4_ was systematically studied using molecular simulation. Hence, this research demonstrates an approach to realizing simultaneous temporal and spatial control of reactions and to overcoming the trade-off between permeability and solute-solute selectivity. It provides insights into the design of highly ultra-permeable and selective NF membranes in water purification, desalination, and resource recovery.

## Results and discussion

### Temporal and spatial modulation of reaction by g-C_3_N_4_

**Fig. 1:**
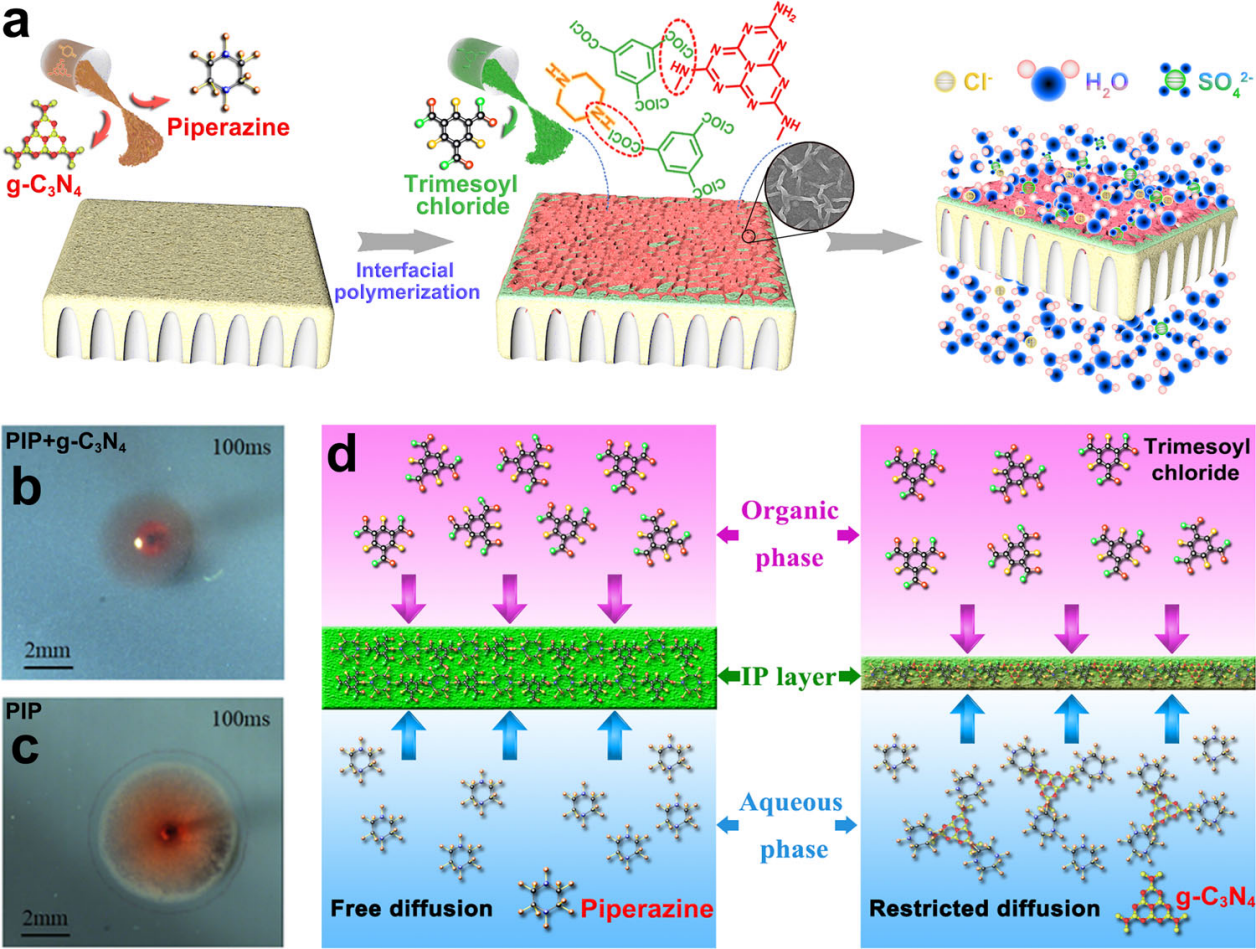
Preparation of NF membranes regulated by g-C_3_N_4_. **a** Schematic illustration of the g-C_3_N_4_-regulated membrane preparation process. **b** Optical photograph capturing the PIP + g-C_3_N_4_ + TMC reaction at 100 ms. **c** Optical photograph capturing PIP + TMC reaction at 100 ms. **d** Illustrations comparing the free and restricted diffusion of PIP during the IP process with and without g-C_3_N_4_.

The key to achieving an ordered structure for ultra permeability and precise separation is to control the IP process, including diffusion and reaction. Here, Fig. 1 illustrates the reaction principle of PIP and g-C_3_N_4_ nanosheet (see Supplementary Fig. 3 and 4) with TMC for preparing the membranes. In the IP reaction process (see Supplementary Fig. 5), amines are dissolved in water, and acyl chlorides are dissolved in an organic solvent. The functional layer forms (Fig. 1a) on top of a porous PES support (see Supplementary Fig. 6) or as a freestanding film (see Supplementary Figure 7) after the two immiscible phases’ contact.

The diffusion coefficient of PIP is about 10^−10^ m^2^ s^−1^ by nuclear magnetic resonance (NMR) measurement. When g-C_3_N_4_ was added into the PIP solution, the monomer diffusivity decreased to 10^−11^ m^2^ s^−1^, one order of magnitude lower than its original value. By real-time online optical monitoring (see Supplementary Fig. 8), it is observed that the spreading area of the (PIP + g-C_3_N_4_)/TMC system (Fig. 1b) is smaller than that of the PIP/TMC system within the same time (Fig. 1c), which confirms that the mobility of PIP within the (PIP + g-C_3_N_4_)/TMC system is lower than that with the PIP/TMC system. Hence, the diffusion rates of the amino monomers may be adjusted by tuning the g-C_3_N_4_ to reach an ideal difference in the diffusivities of monomers in both the aqueous and organic phases^29^ (Fig. 1d).

**Fig. 2:**
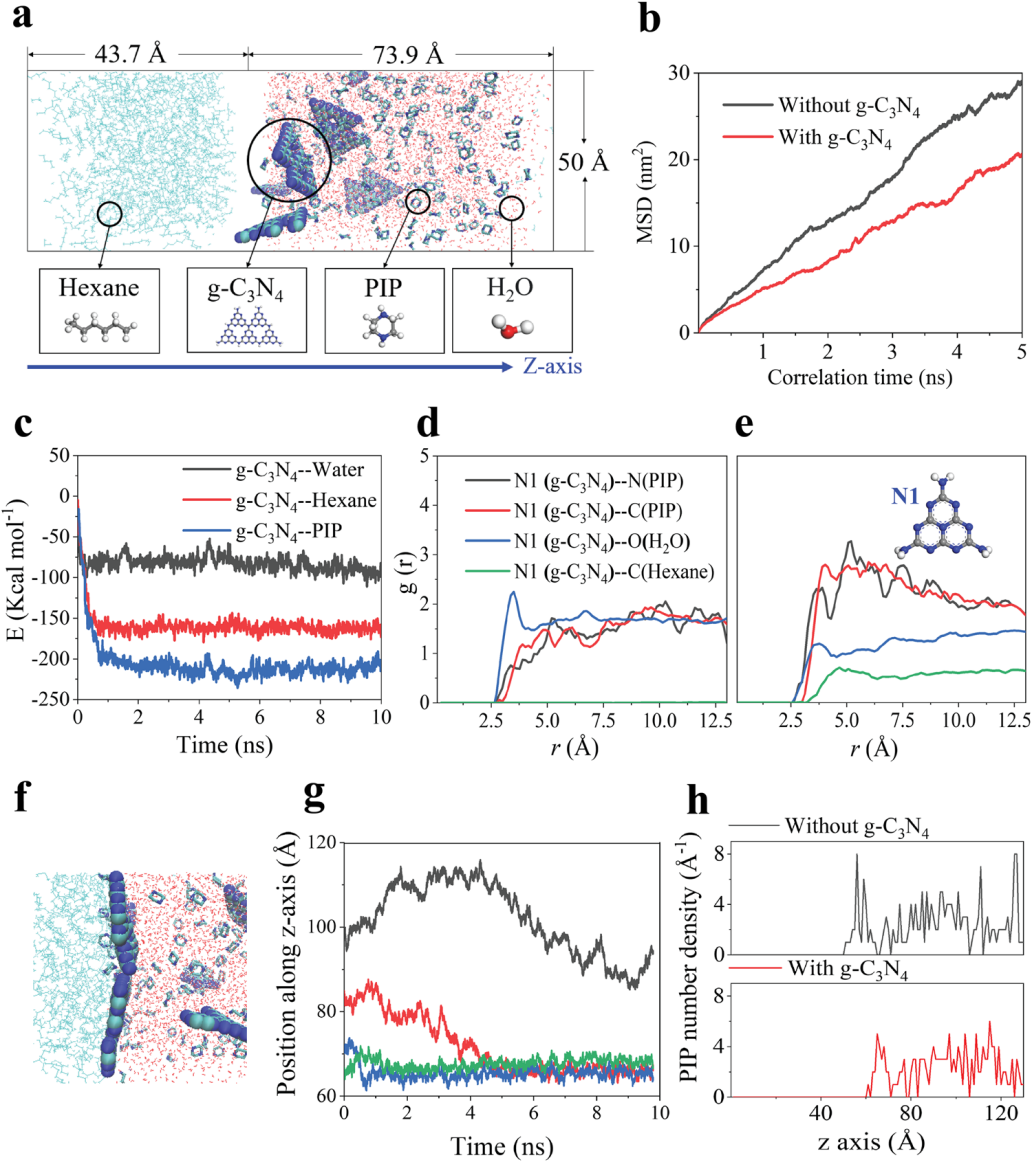
Molecular dynamics simulation of the interfacial polymerization process. **a** Simulation models: the left chamber contains hexane and the right chamber is filled with water, PIP molecules, and g-C_3_N_4_; a similar model without g-C_3_N_4_ was also constructed as control. **b** Time dependence of MSD curves of PIP molecules. **c** Interaction energy between g-C_3_N_4_ and other species calculated by model B (see Methods). **d** The RDF of N1 atoms in g-C_3_N_4_ around PIP (N, C), water (O), and n-hexane (C) at the start of the simulation. **e** The RDF of N1 atoms in g-C_3_N_4_ around PIP (N, C), water (O), and n-hexane (C) at the end of simulation; **f** Image capturing the tiling of g-C_3_N_4_ at the water-hexane interface at the end of the simulation. **g** Time dependence of the position of four pieces of g-C_3_N_4_ nanosheets along the *z*-axis. **h** the PIP number density along the *z*-axis in the simulation systems. Z-axis starts from the hexane phase to the water phase, as is captured in 2a. Source data are provided as a Source Data file.

MD simulations were performed to better understand the roles of g-C_3_N_4_ during the formation of polyamide thin film (see Supplementary Fig. 1). Figure 2a captures model A, which consists of a hexane phase, and an aqueous phase containing water, PIP, and g-C_3_N_4_ nanosheets. A control model without g-C_3_N_4_ was also constructed. The diffusivity of PIP molecules was qualitatively determined by their mean square displacement (MSD) during simulation since the slope of the linear regression between MSD and simulation time is proportional to the dynamic movement of PIP^34^. Figure 2b shows that PIP diffusion significantly decreases in the presence of g-C_3_N_4_. This might be partially explained by the strong affinity between g-C_3_N_4_ and PIP, which is evidenced by the lowest interaction energy between g-C_3_N_4_ and PIP compared to that with water or n-hexane in Fig. 2c, calculated based on Model B (see Supplementary Fig. 9). It is also noteworthy that g-C_3_N_4_ has a higher tendency to interact with n-hexane rather than water. The radial distribution functions (RDF, *g*(r)) of one representative atom in g-C_3_N_4_, named N1, around different atoms in PIP, water, and n-hexane at the start (Fig. 2d) and end (Fig. 2e) of the simulation provide further insights. Starting from a random status, the peak height for N1–H_2_O is reduced, and N1 becomes closely surrounded by the nitrogen atoms in PIP at the end of the simulation, likely through hydrogen bonding. A similar trend is observed with the C atom in g-C_3_N_4_ (see Supplementary Fig.10). It suggests that the hydrogen bonding between g-C_3_N_4_ and PIP may contribute to strong interactions, thus retarding the diffusion of PIP, which is in agreement with experimental observations. This will subsequently lead to better temporal control of the IP process.

In addition, Fig. 2d shows that though originally N1 stays too far away from hexane to be captured by the measurement, they get as close as N1-water at the end. This is evidenced by an interesting tiling behavior of nanosheets at the water-hexane interface, as presented in Fig. 2f. Due to their preferential interaction with hexane over water, the g-C_3_N_4_ nanosheets migrate quickly to the interface along the *z*-axis (Fig. 2g) and pave the interface as flooring tiles. Furthermore, the edges of g-C_3_N_4_ could be decorated with some amine groups, which is proven by the X-ray photoelectron spectroscopy (XPS) result in Supplementary Figure 4b and also captured in its structure in MD simulation. The nanosheets may therefore react with TMC to form a relatively solid ‘floor’. As a result, PIP can diffuse from the water phase to the interface through the slit between adjacent nanosheets or the inter-plane pores. Both the close PIP-g-C_3_N_4_ interactions and interface tiling slow down the diffusion of PIP, leading to a much lower PIP concentration at the interface (Fig. 2h). Tiling also affects the movement of PIP in space, thus controlling the reaction spatially.

### Polyamide membranes with nanoscale-ordered hollow structure

**Fig. 3:**
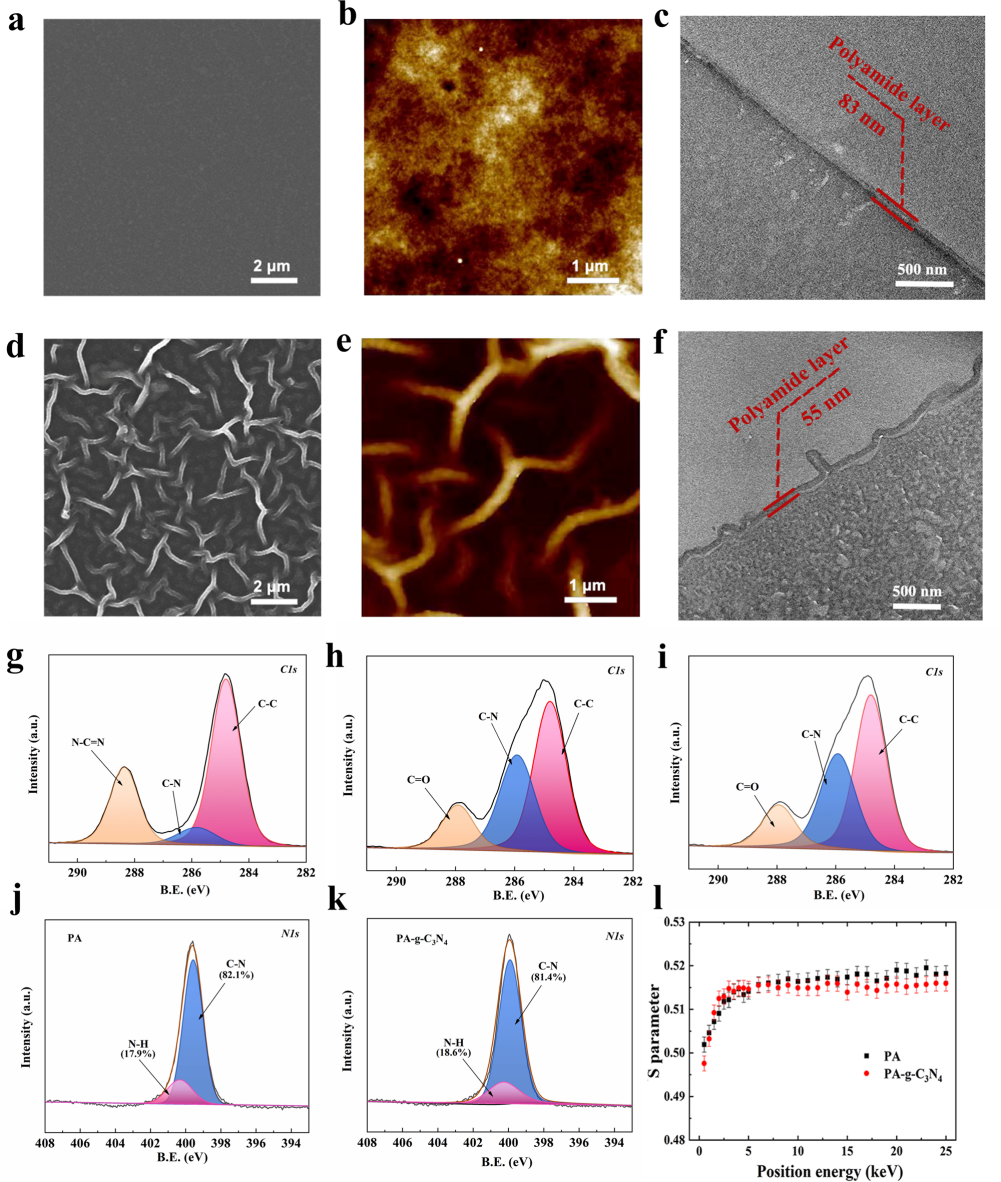
Characterizations of NF membranes. **a** The SEM surface morphology of the PA membrane. **b** The AFM image of the PA membrane. **c** The TEM morphology of PA membrane. **d** The SEM surface morphology of the PA-g-C_3_N_4_ membrane. **e** The AFM image of the PA-g-C_3_N_4_ membrane; **f** The TEM morphology of the PA-g-C_3_N_4_ membrane. **g**–**i** C1s XPS spectra for g-C_3_N_4_, PA, and PA-g-C_3_N_4_ membrane respectively. **j**, **k** N1s XPS spectra for PA and PA-g-C_3_N_4_ membrane respectively. **l** S parameter as a function of positron energy for PA and PA-g-C_3_N_4_ membrane. The concentration of g-C_3_N_4_ for membrane preparation was 0.135 wt%. Source data are provided as a Source Data file.

Figure 3 presents the membrane morphology and properties. The PIP-TMC polyamide membrane surface is smooth (Fig. 3a–c). By adjusting g-C_3_N_4_, nanoscale-ordered hollow structures (Fig. 3d–f, Supplementary Figs. 11 and 12) are obtained. The emergence of ordered structure might be attributed to the retarded diffusion of PIP in the presence of g-C_3_N_4_, similar to the local activation and lateral inhibition phenomenon reported in earlier studies^23,35,36^. However, it means that the number of arched channels increases as g-C_3_N_4_ content rises until reaching 0.135 wt% (Supplementary Figures 12). This is well explained by the tiling effect of nanosheets. PIP diffuses into the surface through the gap between adjacent nanosheets and reacts with TMC forming a hollow structure above the nanosheets where the PIP concentration is lean. With more nanosheets at the interface, the gap density is higher, and its length is shortened. Consequently, the density and size of arched channels are changed. 0.135 wt% of g-C_3_N_4_ is found to give the most uniform structure with a hollow channel. Further increment in nanosheet content may lead to extensive stacking of nanosheets and disturb the ordering of structure. As revealed by the transmission electron microscopy (TEM) image of the membrane cross-section in Fig. 3f, the microscopically arched channels on the surface are hollow. By atomic force microscope (AFM) measurement, the surface area of the PIP/g-C_3_N_4_-TMC layer is found to be 1.76 times that of the PIP-TMC layer (see Supplementary Fig. 13). In the meantime, it is noticed that the thickness of PIP/g-C_3_N_4_ -PA membrane is 55 ± 3 nm, much lower than the control membrane (83 ± 5 nm). Overall, the addition of g-C_3_N_4_ regulates polyamide formation both temporally and spatially, giving a thin, and nanoscale-ordered hollow structure on the surface.

The surface physiochemical properties were then characterized. XPS results in Fig. 3g–i and Supplementary Fig. 14 show that the characteristic N-C = N peak of g-C_3_N_4_ is absent from PA-g-C_3_N_4_ membrane, the N–H bond ratio scarcely changes (Fig. 3j, k), suggesting that g-C_3_N_4_ reside mostly at the bottom of the polyamide layer. It is also noticed the chemical composition and crosslinking degrees of both membrane surfaces are comparable, though PA-C_3_N_4_ has a slightly lower crosslinking degree (Supplementary Tables 4 and 5). Both PA and PA-g-C_3_N_4_ membranes are negatively charged at pH > 3, with the PA-g-C_3_N_4_ membrane being slightly more negative (see Supplementary Fig. 15). Noticeably, the isoelectric points are <pH 3, lower than most other NF membranes, likely due to the high TMC concentration of 0.4 wt% in the reactions that leaves abundant residual carboxylic groups on the surface. The water contact angle of the membrane is reduced to 24.1° (see Supplementary Fig. 16) due to its higher surface roughness. XRD patterns of the PA-g-C_3_N_4_ membrane give a d-spacing of 12.4 Å similar to that of the pure PA membrane (12.1 Å) (see Supplementary Fig. 17). The microstructure was also probed by positron annihilation spectroscopy (PAS). The *S* parameter (Fig. 3l) quickly rises in PA-g-C_3_N_4_ until reaching a plateau, giving a lower selective layer thickness than that of the PA membrane. The slightly lower *S* parameter of PA-g-C_3_N_4_ than the control membrane in the dense layer is likely due to the hindrance of positron diffusion by the g-C_3_N_4_ nanosheet.

### Fast permeation and precise ion-ion separation

**Fig. 4:**
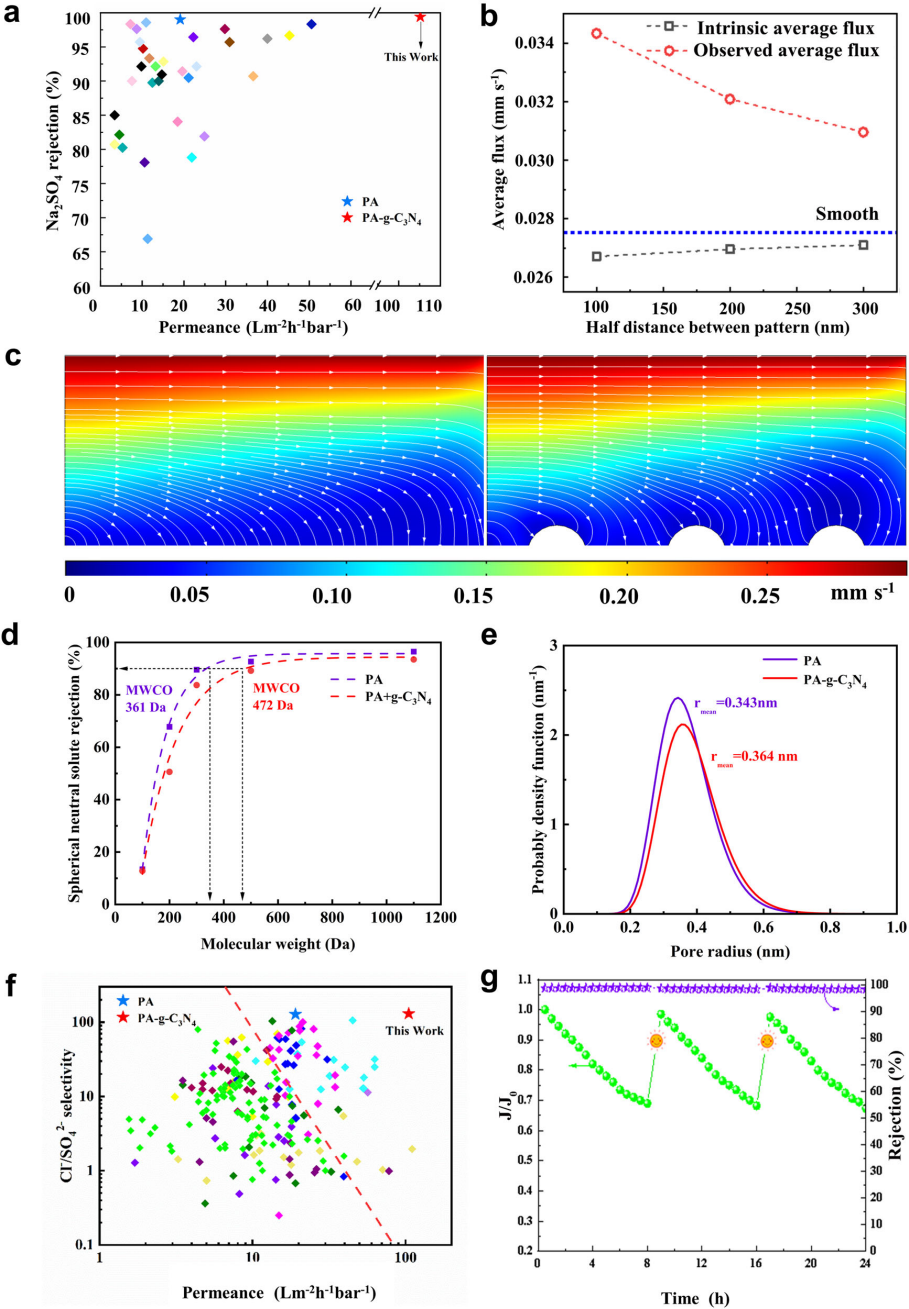
Separation performances of NF membranes. **a** Performance comparison of PA-g-C_3_N_4_ with state-of-the-art NF membranes. Literature data are obtained from ref. ^3^. **b** Average flux of different half distances between patterns. **c** Comparison of the flow streamlines of smooth and nanoscale ordered structure. **d** MWCOs of the PA and the PA-g-C_3_N_4_ membrane. **e** Mean pore size of the PA and the PA-g-C_3_N_4_ membrane. **f** Trade-off between Cl^−^/SO_4_^2−^ selectivity and water permeability of different NF membranes. Literature data are obtained from ref. ^14^. **g** Flux decline and rejection of the PA-g-C_3_N_4_ membrane with methylene blue as the feed solute; cleaning was done with visible light. Source data are provided as a Source Data file.

In the NF test, the PA-g-C_3_N_4_ membrane exhibits water permeance of 105 L m^−2^ h^−1^ bar^−1^, five times higher than that of the PA membrane, while at comparable Na_2_SO_4_ rejection of 99.4% (Fig. 4a). It outperforms state-of-the-art membranes in the permeance-rejection trade-off plot. To get more insights, we performed computational fluid dynamics (CFD) studies (see Supplementary Fig. 2). Figure 4b shows that more transport pathways are found on the curvature of the nanoscale ordered surface leading to higher observed flux. It is consistent with the flow streamlines of smooth and nanoscale-ordered structures (Fig. 4c). However, if flux is normalized against the actual surface area, the intrinsic flux is, in fact, lower than that of smooth membranes. It could be related to the lower pressure drop rate near the surface of the arched structure (see Supplementary Figs. 24–27). Our analysis suggests that the nanoscale-ordered structure enhances water permeation via increased surface area. AFM characterization earlier reveals a ~1.76 times more surface area of the PA-g-C_3_N_4_ membrane, which partially explains its high permeance. In addition, the lower thickness resulting from the temporal and spatial effect of g-C_3_N_4_ also makes a substantial contribution.

Figure 4d shows that the MWCO of the PA-g-C_3_N_4_ membrane is 472 Da, which is slightly larger than that of the PA membrane. A narrow pore size distribution with a pore radius of 0.364 nm is achieved, which lies right between the sizes of hydrated Cl^−^ ions (0.332 nm) and SO_4_^2−^ ions (0.379 nm) (Fig. 4e). Earlier, it is revealed that the membrane surfaces are highly negatively charged. The membrane is thus able to achieve high selectivity of 130 for Cl^−^ over SO_4_^2−^ (Fig. 4f) through both size sieving and Donnan exclusion effects. The Cl^−^/SO_4_^2−^ selectivity remains almost the same in the mixed salt tests with 2000 ppm of salts at varying Cl^−^:SO_4_^2−^ weight ratios (Supplementary Fig. 18).

An additional benefit of our PA-g-C_3_N_4_ membrane is its self-cleaning property. A 100 ppm methylene blue (MB) solution was selected as the model sample to explore the photocatalytic performance under visible light. As shown in Fig. 4g, rejection remains steady at 98.4%, while flux keeps declining due to fouling, The flux can be recovered to 98.1% after three times recycling upon photocatalytic treatment. Supplementary Fig. 20 demonstrates that the PA-g-C_3_N_4_ membrane is much cleaner than the bare PA membrane after cleaning, and supplementary Figure 21 also signifies the important role of photocatalytic cleaning compared to soaking and rising. The mechanism of photocatalytic degradation of MB is illustrated in Supplementary Fig. 22. Electrons (e^-^) and holes (h^+^) are generated by visible light that partially penetrates through the polyamide layer to g-C_3_N_4_^37^, which then react with dissolved oxygen (O_2_) to form superoxide radical anion O_2_^−^^38^. MB is degraded by h^+^ and O_2_^−^ to form CO_2_ and H_2_O, and thus membrane surface becomes clean again. Light is feasible for the PA-g-C_3_N_4_ membrane (see Supplementary Figure 23). This self-cleaning property shall simplify membrane cleaning operation, offering promises for practical application of the g-C_3_N_4_-based NF membrane in textile water purification and other applications where fouling is a concern. The separation performance of the PA-g-C_3_N_4_ membrane is stable for continuous operation (see Supplementary Fig. 19).

In summary, we have fabricated an ultra-permeable, high ion–ion selectivity and self-cleaning g-C_3_N_4_ hybridized NF membrane with a water flux of 105 L m^−2^ h^−1^ bar^−1^ at a 99.4% rejection to Na_2_SO_4_, and a Cl^−^/SO_4_^2−^ selectivity of 130. It represented one of the most competitive overall performances among the reported polymeric NF membranes. This study gives the strategy to tune the nanoscale ordered structure by g-C_3_N_4_ and reveals the role of such structure in transport. The technology offers opportunities for fast and precise separation.

## Methods

### General

Materials used in this work are provided in Chemicals and Materials in Supplementary Materials.

### Preparation of freestanding nanofilms

The 2.0 g PIP, 0.5 g trimethylamine (TEA), and 0.135 wt% g-C_3_N_4_ were dissolved in 100 mL water. Secondly, 0.16 mL phosphoric acid was added to adjust the solution pH to 9. Next, a 0.4 wt% TMC n-hexane solution was prepared. Then, the PIP solution was poured into a petri dish. After that, the TMC organic solution was gently added on top of the aqueous solution to induce IP. After 5 min, a freestanding film was formed that was taken out of the petri dish, washed with DI water three times, and stored in DI water.

### Preparation of PA and PA-g-C_3_N_4_ membranes

The PA and PA-g-C_3_N_4_ NF composite membranes were prepared by IP on top of the polyether sulfone (PES, MWCO 100 kDa) ultrafiltration (UF) membranes. The aqueous solution was prepared with 0.2–2.0 g of PIP and 0.5 mL TEA in 100 mL water, and its pH was tuned to 9 by adding 0.16 mL phosphoric acid. To prepare the PA-g-C_3_N_4_ composite membrane, g-C_3_N_4_ nanosheet was added at the concentrations of 0.075 to 0.145 wt% to form well-dispersed suspensions. The organic solution was prepared by dissolving 0.1–0.5 wt% TMC in n-hexane. Then, the PES membrane was dipped into the aqueous phase for 5 min. After being taken out, the excess solution on its surface was removed using a rubbery roller, and the support was left at room temperature until the surface appeared dull and dry. Next, the membrane was immersed in the organic phase for 1 min. After that, the membrane was heated in an oven at 80 °C for 5 min, and stored in DI water before use. Three replicate membranes for each experiment were fabricated and examined. Error bars represent the standard deviation of the three membranes.

### Characterization

SEM images were taken by a Hitachi S4800 cold field emission scanning electron microscopy. TEM images were conducted by an FEI Tecnai G2 F20 S-TWIN 200KV field-emission transmission electron microscopy. The elemental composition of the membrane surface was analyzed by an X-ray photoelectron spectrometer (XPS, Thermo ESCALAB250Xi, USA). XRD was tested by a Bruker AXS D8 Advance powder X-ray diffractometer. AFM images were measured by NanoScope MultiMode scanning probe microscopy (Veeco, Camarillo, California, US).

### Membrane performance test

The permeation performance of the membrane was measured on the cross-flow filtration equipment. The effective area of the membrane was 26 cm^2^. The solution temperature was maintained at 25 °C by a heat exchanger. In order to achieve a steady state, the membranes were pre-pressurized for 2 hours under 6 bar. The flow rate was 1.5 L min^−1^. The concentrations of salts (including single NaCl and Na_2_SO_4_) and dyes in feed solutions were 2000 ppm and 100 ppm, respectively.

The water permeance was calculated based on Eq. 1.

1 $$L=\frac{V}{A\cdot \Delta t\cdot \triangle p}$$

where *L* is the water permeance (L m^−2^ h^−1^ bar^−1^), *V* (m^3^) is the volume of permeate collected over Δ*t, A* is the effective membrane area (m^2^), Δ*t* and Δ*p* represent the filtration time (h), and the transmembrane pressure.

2 $$R=\left(1-\frac{{C}_{p}}{{C}_{f}}\right)\times 100\%$$

where *R* is the salt rejection (%), *C*_*p*_ and *C*_*f*_ represent the concentration of permeate and feed solutions, respectively. The salt concentration was quantified by conductivity measurement, and dye solutions were measured by UV–vis.

In addition, mixed salt tests were performed in a similar way using the mixed solutions of NaCl and Na_2_SO_4_, where the total concentrations were kept constant at 2000 ppm while the weight ratios of Cl^−^:SO_4_^2−^ were varied. The Cl^-^ and SO_4_^2−^ concentrations were measured by ion chromatography.

The Cl^−^/SO_4_^2−^ selectivity was calculated based on the following equation:

3 $${S}_{{{{{{\rm{C}}}}}}{1}^{-}/S{{O}_{4}}^{2-}}=\frac{1-{R}_{{{Cl}}^{-}}}{1-{R}_{S{{O}_{4}}^{2-}}}$$

### MD simulation

MD simulations in this work were conducted using the large-scale atomic/molecular massively parallel simulator (LAMMPS)^39^ package on a parallel Linux cluster. The results were visualized using the VMD^40^, and the initial setup was constructed with the Materials Studio 8.0 software. Water molecules were modeled using the SPC potential^41^, whereas other molecules were characterized using the OPLS–AA force field^42^, the parameters for which are shown in Supplementary Table 1. For all dynamics runs, the temperature was controlled using the Nosé–Hoover thermostat, and the pressure was controlled with the Berendsen barostat. The Lennard-Jones (LJ) and coulomb potentials were combined to compute pair interactions between atoms. The particle–particle–mesh (PPPM) k-space solver^43^ was used to address the long-range columbic interactions with a relative accuracy of 10^−6^. Two computing models were constructed in this work. Model A in Fig. 2a composes of an n-hexane phase and a water phase containing PIP molecules. The number of molecules and the cell dimensions can be found in Supplementary Table 2. A comparative system was likewise constructed with the addition of g-C_3_N_4_. The densities of water and n-hexane, as well as the concentrations of PIP, were determined in accordance with ref. ^44^. Throughout the simulation, the SHAKE algorithm^45^ was used to constrain the bond and angle of water molecules. Initially, energy minimization was utilized to eliminate atom overlaps. Following that, a 10 ns NVT run (*T* = 300 K) was performed to obtain data for further analysis. The non-bonded interaction energy between g-C_3_N_4_ and the other species was determined using Model B. A g-C_3_N_4_ layer was put at the bottom of the simulation cell, and water, n-hexane, and PIP molecules were deposited on it independently in each of the three simulation cells; the interaction energy between them and the g-C_3_N_4_ layer was determined after equilibrium had been attained.

## Supplementary information

Supplementary Information

Peer Review File

## Data Availability

The authors declare that the data supporting the findings of this study are available within the paper and its supplementary information file. Source data are provided in this paper. All data are also available by request to the corresponding authors. Source data are provided in this paper.

## Source data

Source Data
