# Supplementary material for: Polyamide membranes with nanoscale ordered structures for fast permeation and highly selective ion-ion separation
Source: Nat Commun. 2023 Feb 27;14:1112. doi: 10.1038/s41467-023-36848-8 (PMC9971196; doi:10.1038/s41467-023-36848-8)
Supplement: Supplementary file 1 — Supplementary Information [file 41467_2023_36848_MOESM1_ESM.pdf]

Supplementary Information for

Polyamide Membranes with Nanoscale Ordered Structures for Fast Permeation and Highly Selective Ion-ion Separation

Changwei Zhao[1#,*], Yanjun Zhang[1#], Yuewen Jia[2#], Bojun Li[3#], Wenjing Tang[1], Chuning Shang[2], Rui Mo[1], Pei Li[4], Shaomin Liu[4], Sui Zhang[2*]

[1] *College of Resources and Environmental Sciences, China Agricultural University, Beijing 100193, China.*

[2] *Department of Chemical and Biomolecular Engineering, National University of Singapore, 4 Engineering Drive 4, 117585, Singapore*

[3] *State Key Laboratory of Water Environment Simulation, School of Environment, Beijing Normal University, Beijing 100875, China*

[4] *College of Materials Science and Engineering, College of Chemical Engineering, Beijing University of Chemical Technology, 100029 Beijing, China.*

[#] These authors contributed equally to the work

[*] Corresponding authors: zhaocw@cau.edu.cn (C. Zhao)

chezhasu@nus.edu.sg (S. Zhang)

Table of contents

1. Transport properties

1.1. Interfacial polymerization 1

1.2. Transport properties 1

2. Material synthesis and preparation 2

2.1. Chemicals and materials 2

2.2. Synthesis of g-C_3N_4 nanosheet sol solution 2

3. Characterization 3

3.1.Methods and instruments 3

3.2. Molecular dynamics simulation 4

3.3. Computational fluid dynamics (CFD) study 6

3.4. G-C_3N_4 preparation and characterization 8

3.5. Membrane fabrication 9

3.6. Optical observations 11

3.7. Additional MD simulation results 12

3.8. SEM characterization 13

3.9. AFM characterization 14
3.10. Chemical composition by XPS 16
3.11. Zeta potential 16
3.12. Water contact angle 17
3.13. XRD spectra 17
4. Separation performance 18
4.1. Permeation and rejection of salts 19
4.2. The Cl^-/SO_4^{2-} selectivity 21
4.3. Long-term experiment 22
4.4. Anti-fouling behaviour of PA and PA-g-C_3N_4 23
5. CFD results of the permeation through nanoscale ordered structure 27
6. Supplementary References 29

1. Transport properties

1.1. Interfacial polymerization

Interfacial polymerization (IP) process involves at least two types of high reactivity monomers that can react and diffuse at the interface of two immiscible liquid phases far from thermodynamic equilibrium [1-3]. Theoretical approach for IP is to solve the system of differential reaction-diffusion equations based on Eq. S1.

$$\frac{\partial c_i}{\partial t} = r_i(c) + D_i \frac{\partial^2 c_i}{\partial x^2} \tag{S1}$$

where c_i is the local concentration (mol/m^3) of species i in the reaction zone, $\boldsymbol{c}= \{c_i\}$, x is the coordinate across the interface (m), t is the polymerization time (s), r_i and D_i are the reaction rate (mol/m^3·s) and the diffusion coefficient (m^2/s) of the corresponding species.

1.2. Transport properties

The solution-diffusion model was used to describe the transport of water and salt through membranes. The water flux through membrane is following:

$$J_w = A\Delta p \tag{S2}$$

where J_w is the water flux (L/m^2·h), A is the water permeability constant (L/m^2·h·bar), Δp is the

pressure difference across the membrane (bar). The salt flux across membrane is as follows:

$$J_s = B\Delta C_s \quad \text{(S3)}$$

where J_s is the salt flux (mg/m^2·h), B is the salt permeability constant (m/h), and Δc_s is the salt concentration difference.

2. Material synthesis and preparation

2.1. Chemicals and materials

Piperazine (PIP, > 99.0%), Trimesoyl chloride (TMC, > 98.0%), trimethylamine (TEA), *n*-decane (C10H22 > 99.0%) were purchased from Shanghai Aladdin Reagent Company (Shanghai, China). Urea (> 99.0%), Sodium hydroxide (NaOH , > 99.0%), Sodium sulfate (Na_2SO_4, ≥ 99.0%), sodium chloride (NaCl, ≥ 99.5%), magnesium chloride ($MgCl_2$, ≥ 99.5%), magnesium sulfate ($MgSO_4$, ≥ 99.5%) and methylene blue (> 99.0%) were purchased from Sinopharm Chemical Reagents Co. Ltd. (Beijing, China). Polyether sulfone (PES) (molecular weight cut-off: 100 kDa) were provided by Ande Membrane Separation Technology (Beijing) co. Ltd (Beijing, China). They were stored in 1% $NaHSO_3$ aqueous solution and in fresh deionized water before use.

2.2. Synthesis of g-C_3N_4 nanosheet sol solution

A certain amount of urea was used as the precursor, and the light yellow g-C_3N_4 bulk sample was obtained by heating it in a muffle furnace at 550 °C for 4 h. The g-C_3N_4 was dispersed in the solution by sodium hydroxide treatment to improve g-C_3N_4 dispersion and stability. Consequently, the light yellow block sample was grounded into powder. After that, 3M NaOH aqueous solution was added to make a solution of 25 mg/ml, and the solution was heated and stirred at 60 °C for 12 h. The solution was then dialyzed to neutrality with a 3500 Da dialysis bag to obtain a g-C_3N_4 naosheet sol.

Methylene blue adsorption in solution was investigated as follows: PA membrane and PA-g-C_3N_4 membrane were immersed in MB solution (100 ppm, 50 ml) and irradiated under light at different times. In addition, one PA-g-C_3N_4 membrane in MB solution (100 ppm, 50 mL) under dark

conditions was also studied as a control. The UV-Vis absorbance of the solution was measured at the start and end of the experiment.

3. Characterization

3.1.Methods and instruments

The IP process behavior of oil-phase droplet on water-phase droplet was observed by optical microscope. Firstly, drops a certain amount of aqueous solution on the glass, then oil phase was gently injected in the middle of the water droplets. IP process at different times were recorded under optics apparatus. The pulse-gradient spin-echo nuclear magnetic resonance (NMR) was used to measure the diffusion coefficient. The Bruker Avance III 600 spectrometer (Switzerland) recorded the spectra. Ion chromatography (Dionex, ICS-1100, Sunnyvale, CA, USA) was used to measure the ion concentration. The OCA20 contact angle analyzer (DataPhysics Instruments GmbH, Filderstadt, Germany) was used to measure the contact angle. A droplet of 5 μL DI water was delivered onto a membrane surface using a micro syringe, and a static image of the droplet in equilibration with the membrane surface was taken. Contact angle was computed by circle fitting method by drop shape analysis software SCA 20. For any given membrane sample, contact angles were measured five different locations to evaluate the average value.

MWCO is determined by the retention rate of spherical neutral solutes selected for retention reactions with different PEG molecular weights. By calculating the Stokes radius of the spherical solute of interest and neglecting the effect of steric and hydrodynamic interactions between solute and pore space on solute rejection, the pore size distribution of the NF membrane can be expressed as the following probability density function.

$$\frac{dR(r_p)}{dr_p} = \frac{1}{r_p \, ln\sigma_p \sqrt{2\pi}} exp\left[-\frac{(lnr_p - ln\mu_p)^2}{2(ln\sigma_p)^2}\right] \qquad (S4)$$

where μ_p is the average pore size of the composite membrane, μ_p is the Stokes radius of the spherical solute corresponding to a rejection rate of 50.0%, σ_p is the ratio between the Stokes radius with a rejection of 84.13% to the Stokes radius with a rejection of 50.0%, r_p is the Stokes radius of the solutes.

3.2. Molecular dynamics simulation

Details of MD simulation is given in the main text. Supplementary Tables 1 and 2 list the parameters. Supplementary Figure 1 shows the simulation model B.

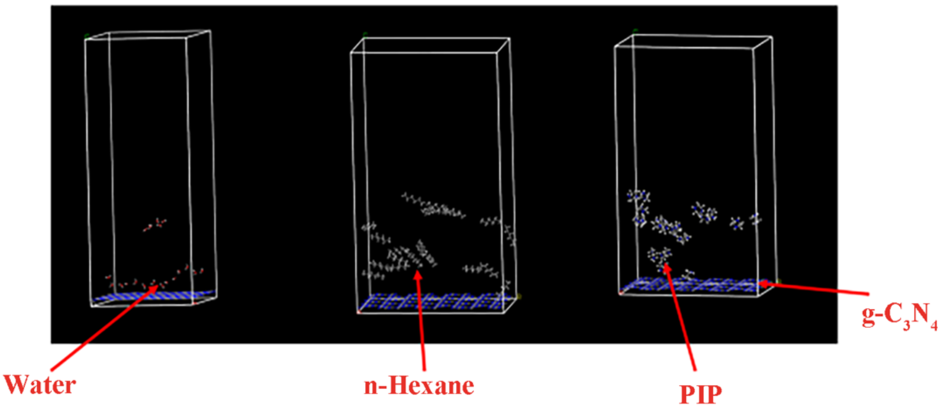

Supplementary Figure 1. Model B that simulates the interaction between g-C_3N_4 and other species including water, n-hexane and PIP.

Supplementary Table 1. Lennard-Jones potentials (OPLS-AA).

	Atom	ε (kcal mol-1)	σ (Å)
n-Hexane	c4	0.066	3.50
	h1	0.015	2.50
PIP	c4	0.066	3.50
	n3h1	0.170	3.30
	h1	0.030	2.50
	h1n	0.000	0.00
g-C_3N_4	n3h2	0.170	3.25
	c3a	0.070	3.55
	n2a	0.170	3.25
	n3a	0.170	3.25

	n3h1	0.170	3.30
	n3	0.170	3.25
	h1n	0.000	0.00

Supplementary Table 2. Molecule number and dimensions of model A.

Number of molecules	H_2O	5000
	n-Hexane	500
	pip	200
	C_3N_4	4
Dimension of the simulation cell (Å)	x	50
	y	50
	z	118

3.3. Computational fluid dynamics (CFD) study

2-D simulation domains for smooth membrane and membranes with hollow structures were generated in our CFD models (Supplementary Figure 2). The radius of the hollow structure (*r*) and membrane thickness (*th*) for simulation were set as 50 and 25 nm, respectively. The half distance (*d*) between hollow structures was varied to explore its influence on membrane flux.

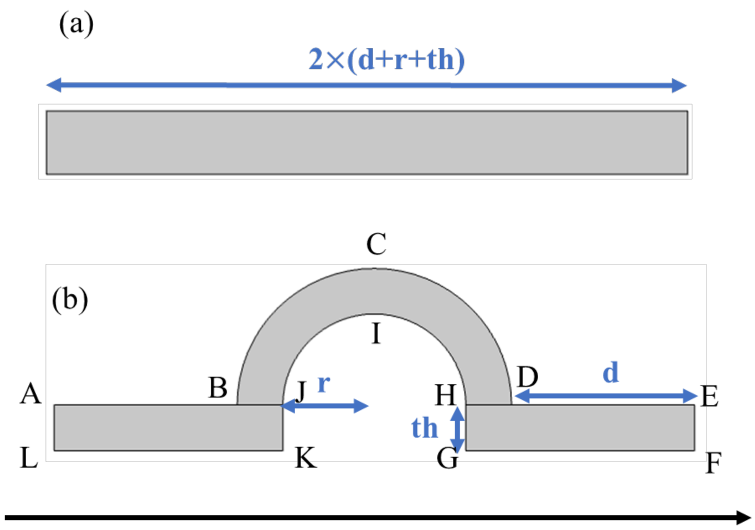

Supplementary Figure 2. (a) Smooth membrane and (b) Membrane with ordered hollow cone structures in simulation.

The water flux in membrane was given by Eq. S5. Therefore, a pseudo-Darcy law was assumed to describe the water transport across the composite membrane:

$$J_w = -\frac{k}{\mu t} \times p \qquad \text{(S5)}$$

where k is the permeability in Darcy's law and μ is the dynamic viscosity of water. $k/\mu t$ was set based on membrane permeability, which was 20 L/m^2 h^{-1} bar^{-1}. The boundary conditions for ABCDE and FGHIJKL were the pressures for inlet and outlet flow (Supplementary Table 3). Symmetrical boundary conditions were also applied to AL and EF. Mesh sensitivity tests have been done prior to the simulation. The number of elements for smooth membrane and membranes with half distance between hollow structures of 100, 200, 300 nm were 248112 and 461358, 659758, 858686 respectively. The relationship between intrinsic and observed flux for each membrane were

calculated based on equations below:

$$J_{w\_obs} = J_{w\_\text{int}} \times \frac{S_{effective}}{S} = J_{w\_\text{int}} \times \frac{2 \times d + \pi \times (r + th)}{2 \times (d + r + th)} \quad \text{(S6)}$$

where S and $S_{effective}$ are the apparent surface area and effective surface area of membranes. Note that apparent surface area refers to the surface area of a smooth membrane, and effective surface area refers to the actual area that contributes to total permeation by taking into consideration of the surface roughness. Membranes with hollow cone structure has a higher effective surface area than the apparent surface area.

Supplementary Table 3. Boundary conditions for CFD simulations. The designations correspond to Figure S2.

Boundary designation	Solute Transport
Inlet (ABCDE)	p_{inlet}=5 bar
Outlet (FGHIJKL)	p_{outlet}=0
Side wall (AL/EF)	Symmetry

3.4. G-C_3N_4 preparation and characterization

Supplementary Figure 3 gave the fabrication process of g-C_3N_4 nanosheet sol. The g-C_3N_4 precursor urea was calcined at high temperature and light yellow layered g-C_3N_4 bulk was obtained, then alkali treatment was performed to obtain a highly dispersed and stable g-C_3N_4 sol solution.

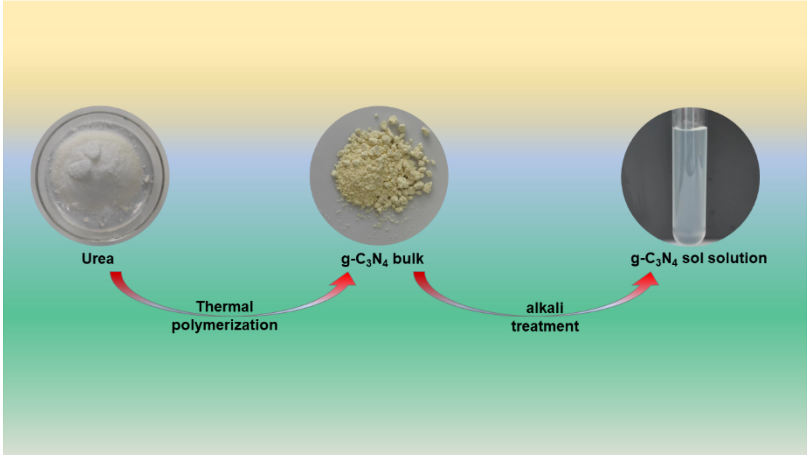

Supplementary Figure 3. Preparation and characterization of g-C_3N_4 nanofibers network sol.

As seen in Supplementary Figure 4a, the g-C_3N_4 material shows the nanosheet structure. The g-C_3N_4 nanosheets were dispersed in the solution by alkaline treatment to improve its dispersibility and stability. The XPS analysis (Supplementary Figure 4b) confirms there are C-N-H, N-$(C)_3$ and C-N=C groups. The g-C_3N_4 nanosheets were well dispersed in water as is proved by the observed Tyndall effect (Supplementary Figure 4c) .

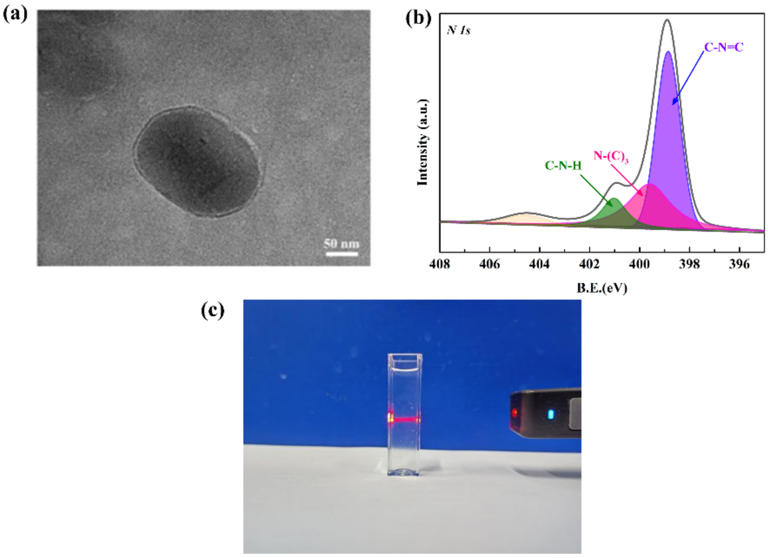

Supplementary Figure 4. (a)The TEM of g-C_3N_4. (b) N*1s* XPS spectra of g-C_3N_4. (c) The Tyndall effect.

3.5. Membrane fabrication

In a typical membrane synthesis, IP of monomeric amine and acid chloride reacted at the surface of an asymmetry porous support to form PA membranes. Supplementary Figure 5 shows membrane chemical structure formed by the reaction between PIP, g-C_3N_4 and TMC. Note that the -N-H groups at the edges of g-C_3N_4 could react with TMC.

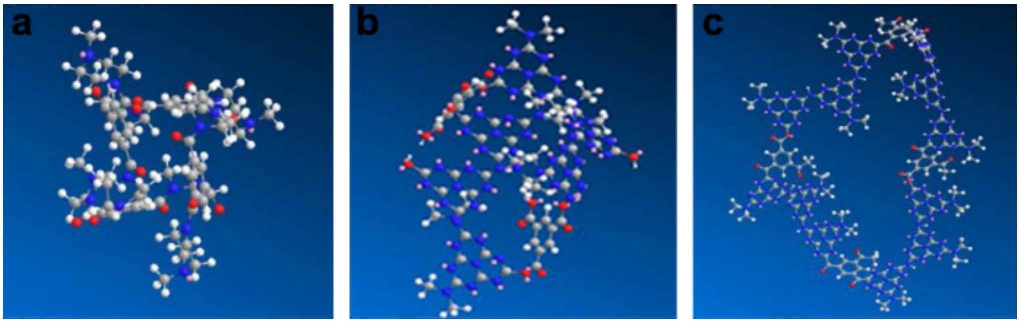

Supplementary Figure 5. (a) Interfacial polymerization of PIP and TMC to form polyamide (PA) membranes. Chemical structures. (b) Simulated structure of the g-C_3N_4+TMC polymer. **(c)** Simulated structure of the (PIP/g-C_3N_4) +TMC polymer. Gray, white, blue, red circles denote C, H, N, O atoms, respectively.

Supplementary Figure 6 compares the cross-section SEM images of the porous PES supports. The porous PES support is finger-like structure with a loose top surface layer.

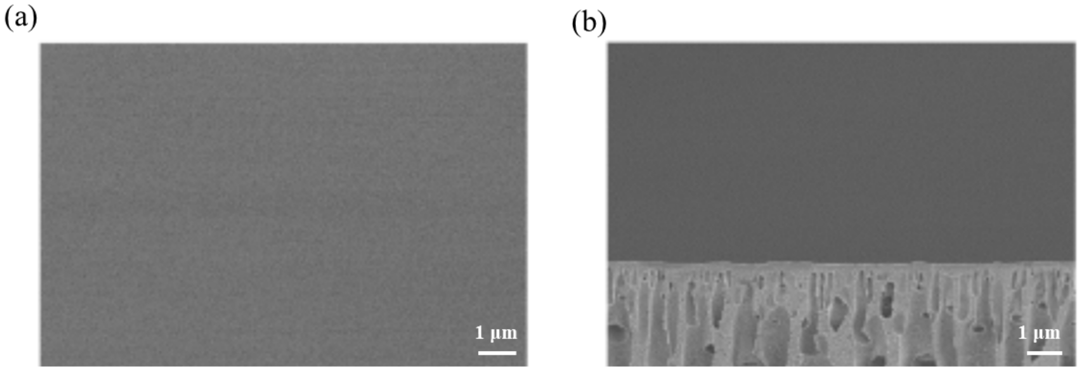

Supplementary Figure 6. (a) Surface image of the PES supports. (b) Cross-section image of the PES supports.

Supplementary Figure 7 shows the photographs of the freestanding PIP, g-C_3N_4 and PIP+g-C_3N_4 membrane. The one with g-C_3N_4 results in the slower membrane form and thinner thickness.

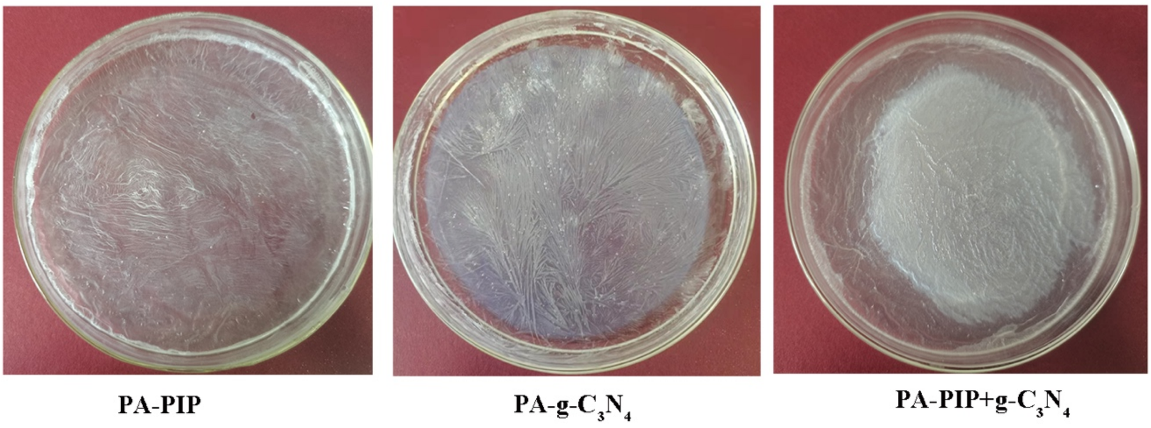

Supplementary Figure 7. The images of free-standing membrane.

3.6. Optical observations

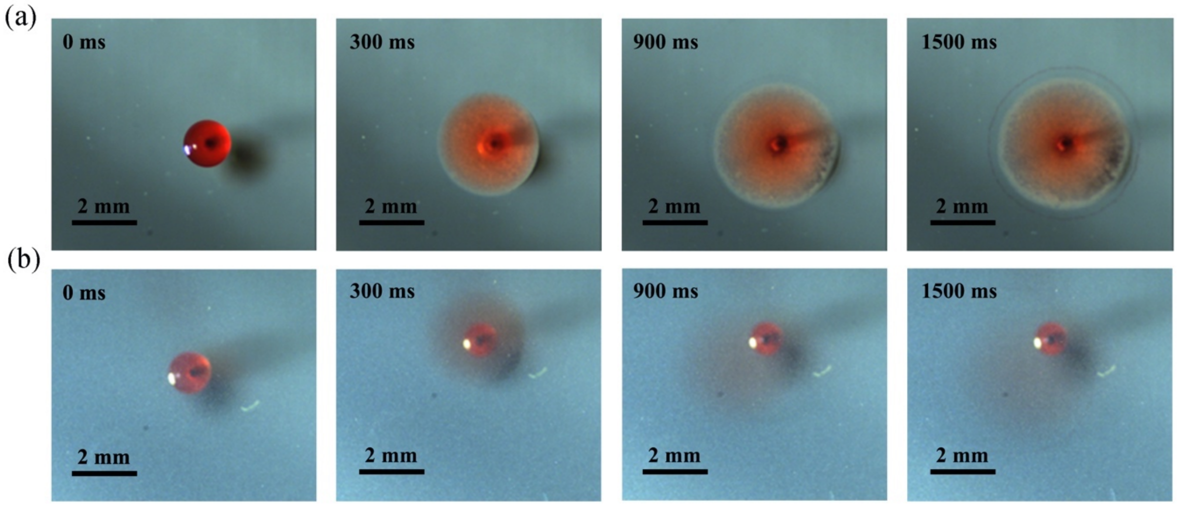

Supplementary Figure 8. Optics images at different reaction time. (a) PIP+TMC reaction process. (b) PIP+g-C_3N_4+TMC reaction process.

3.7. Additional MD simulation results

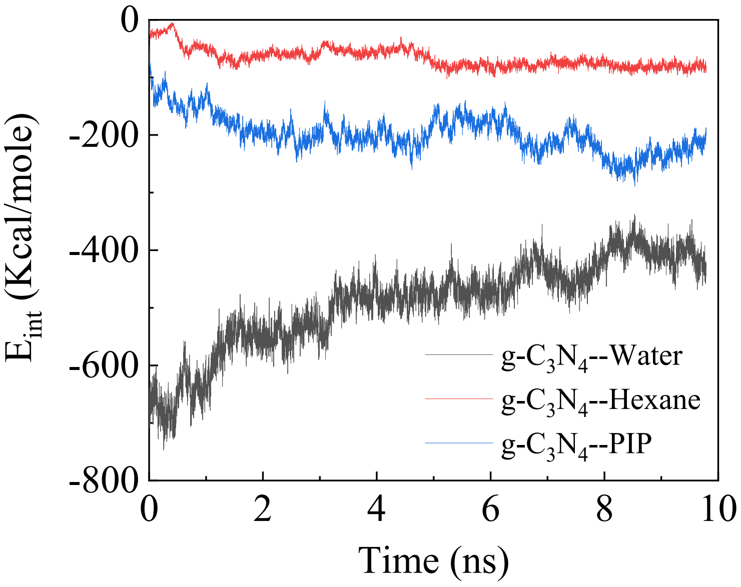

Supplementary Figure 9. Time evolution of interaction energy between g-C_3N_4 and other species in Model A.

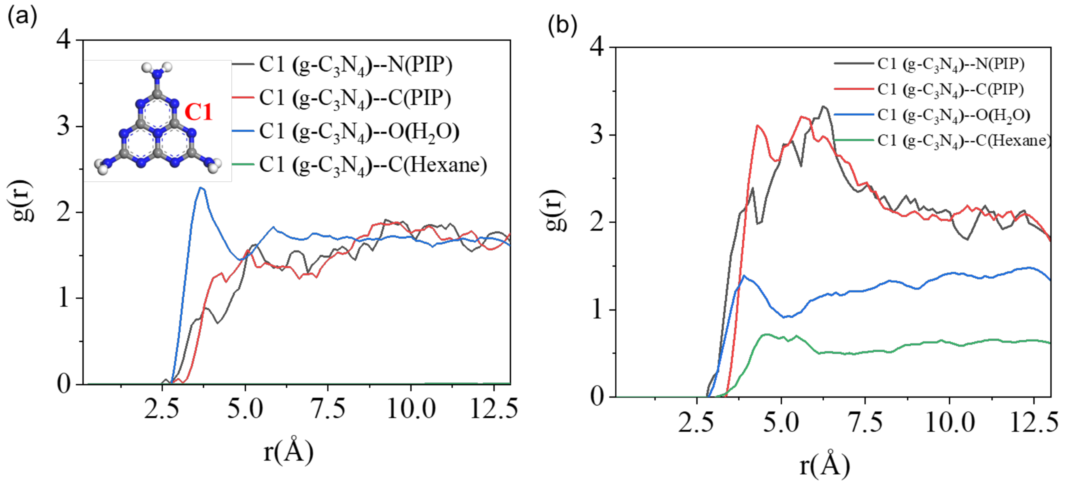

Supplementary Figure 10. The RDF of C1 atoms in g-C_3N_4 around PIP (N, C), water (O), and n-Hexane (C) (a) at the start and (b) The end of simulation.

3.8. SEM characterization

The SEM images of the different TMC concentrations at 0.135 wt% g-C_3N_4 is shown in Supplementary Figure 11, it can be seen that with the increase of TMC concentration till 0.4 wt%, the nanoscale structure becomes more ordered. When TMC concentration reaches 0.5 wt%, the surface becomes less regular.

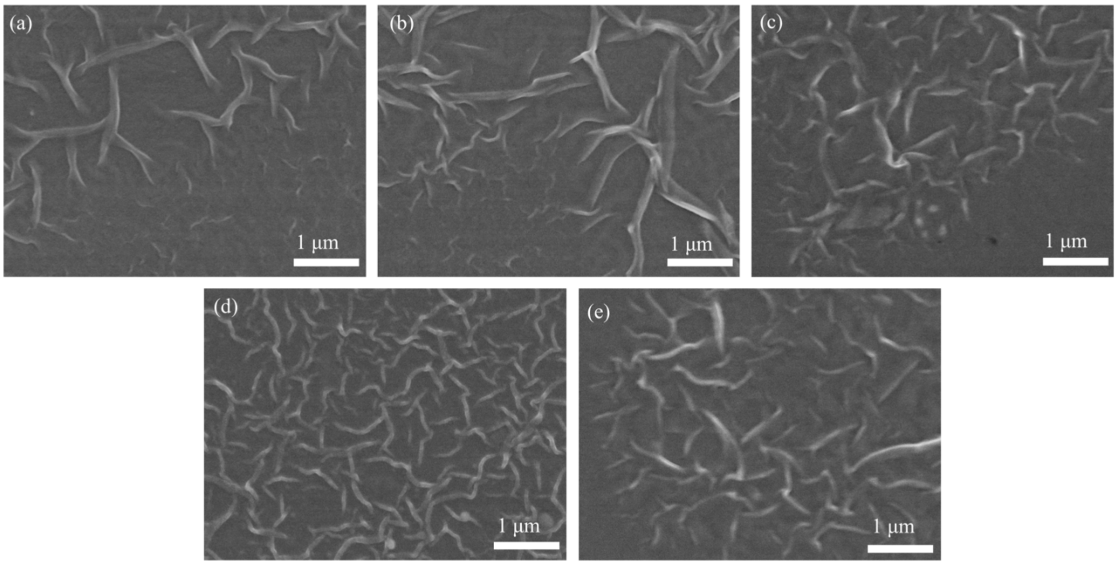

Supplementary Figure 11. SEM surface morphologies of PA-g-C_3N_4 membranes with different TMC concentrations. (a**)** 0.1 wt%. (b) 0.2 wt%. (c) 0.3 wt%. (d) 0.4 wt%. (e) 0.5 wt%.

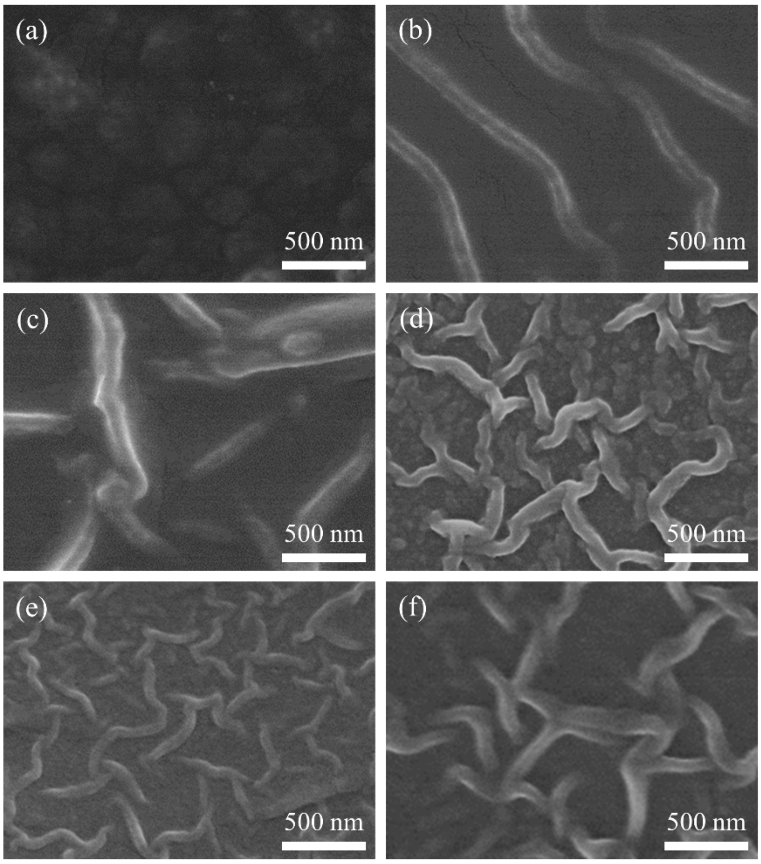

Supplementary Figure 12. SEM surface morphologies. (a) PA membrane. (b) PA-g-C_3N_4 membrane (0.075 wt%). (c) PA-g-C_3N_4 membrane (0.115 wt%). (d) PA-g-C_3N_4 membrane (0.125 wt%). (e) PA-g-C_3N_4 membrane (0.135 wt%). (f) PA-g-C_3N_4 membrane (0.145 wt%).

3.9. AFM characterization

As shown in Supplementary Figure 13, the PA membrane shows a flat surface. The PA-g-C_3N_4 membrane shows nanoscale ordered structure on the membrane surface, which becomes more significant with the increase of g-C_3N_4 content.

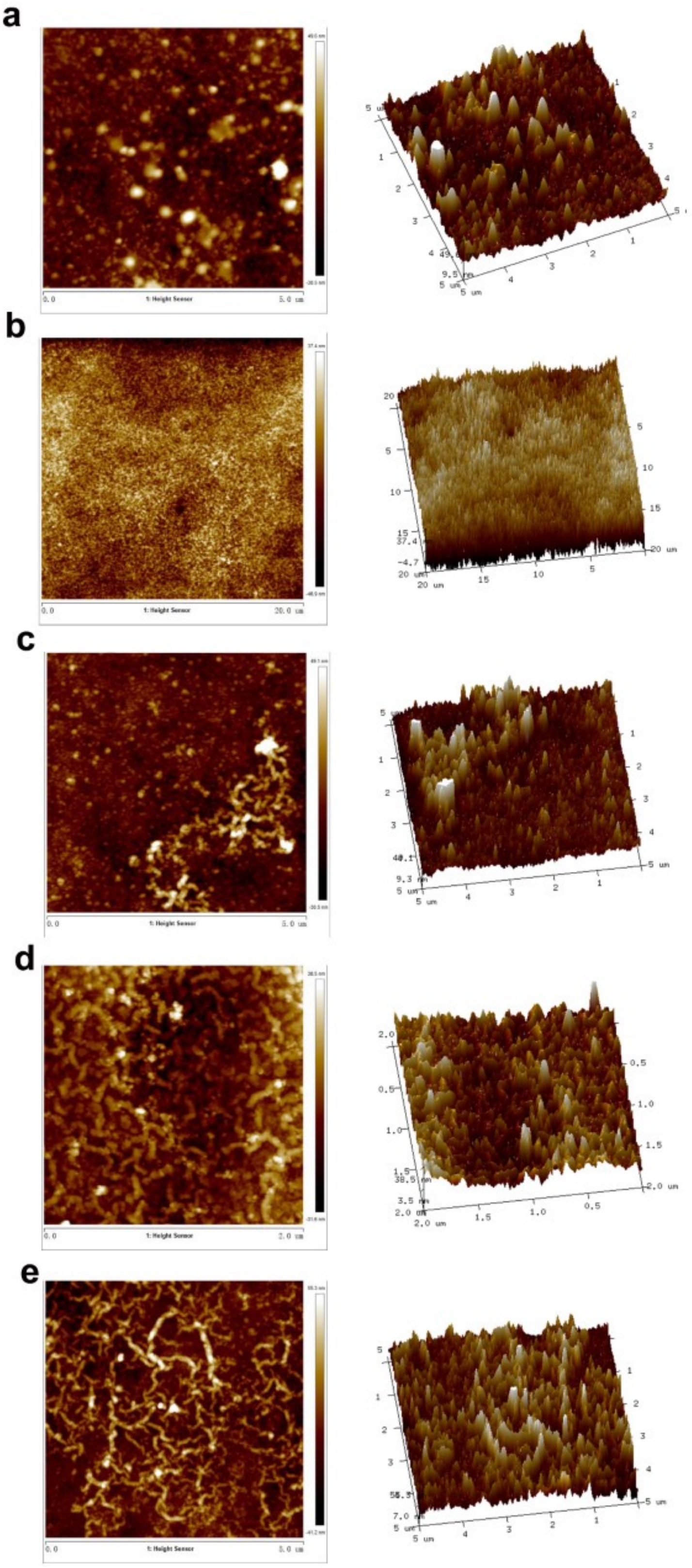

Supplementary Figure 13. AFM morphologies. (a) PA membrane. (b) PA-g-C_3N_4 membrane (0.075 wt%). (c) PA-g-C_3N_4 membrane (0.115 wt%). (d) PA-g-C_3N_4 membrane (0.125 wt%). (e) PA-g-C_3N_4 membrane (0.135 wt%).

3.10. Chemical composition by XPS

XPS widescan spectra for g-C_3N_4 nanosheet and composite NF membranes are provided in Supplementary Figure 14, showing the presence of C, N and O elements on their surfaces.

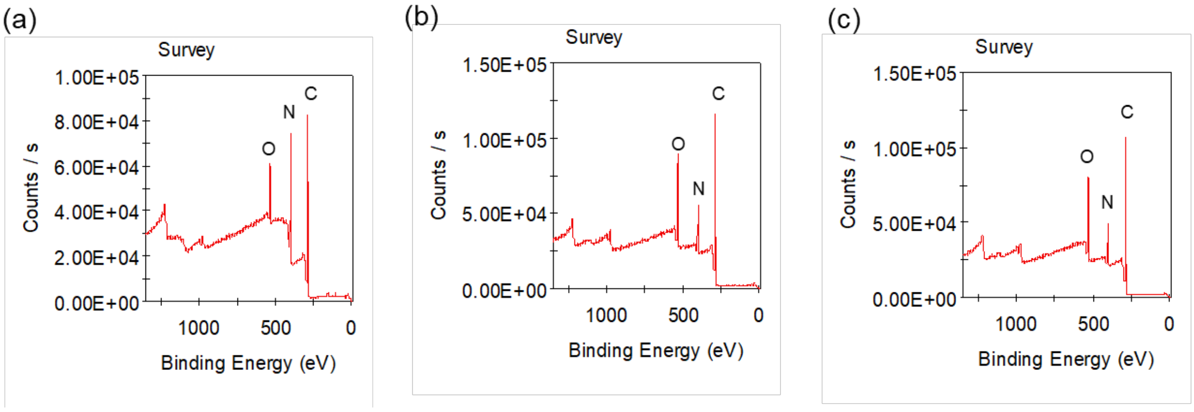

Supplementary Figure 14. The XPS widescan survey spectra of (a) g-C_3N_4. (b) PA and (c) PA-g-C_3N_4

C1s spectra for g-C_3N_4 nanosheet and composite NF membranes are given in the main text. The de-convoluted peaks in C1s spectra are analyzed in Supplementary Table 4. The surface elemental composition is listed in Supplementary Table 5. The relative elemental composition was determined based on the intensity of the C 1s, N 1s, O 1s peaks at 284.6, 397.9, and 531.6 eV, respectively. It can be seen that the g-C_3N_4 addition increased the degree of crosslinking of membrane.

Supplementary Table 4. Analysis of peaks in C1s spectra for g-C_3N_4 nanosheet and composite NF membranes.

Membrane	Atomic percentage corresponding to peak, %			
	C-C (284.8 eV)	C-N (285.9 eV)	C=O (287.9 eV)	N-C=N (288.4 eV)
G-C_3N_4 nanosheet	63.2	8.4	0	28.6
PA membrane	50.3 ± 1.1	35.0 ± 0.5	14.7 ± 0.6	0
PA-g-C_3N_4 membrane	52.1 ± 1.5	33.3 ± 1.9	14.6 ± 0.5	0

Supplementary Table 5. Element content of the composite NF membranes.

Membrane	Surface atomic composition (%)			O/N
	C	N	O	
PA membrane	72.8±1.4	12.1 ±0.2	15.1 ±1.3	1.25 ± 0.09
PA-g-C_3N_4 membrane	73.0 ±0.1	11.5 ±0.1	15.4 ±0.2	1.33 ± 0.03

3.11. Zeta potential

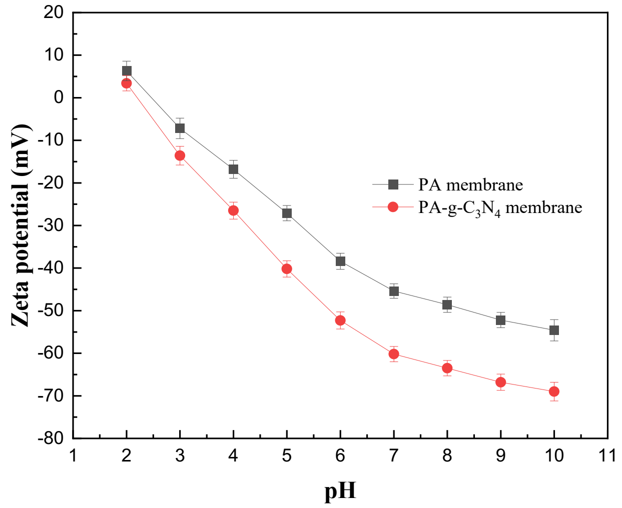

Supplementary Figure 15. Zeta potential of the PA and PA-g-C_3N_4 membranes.

3.12. Water contact angle

The contact angles of PES support and PA membrane were 63.0±0.6° and 39.5±0.5°, respectively. With the g-C_3N_4 addition, contact angle decreased to 24.1±0.3° corresponding to the hydrophilicity improvement.

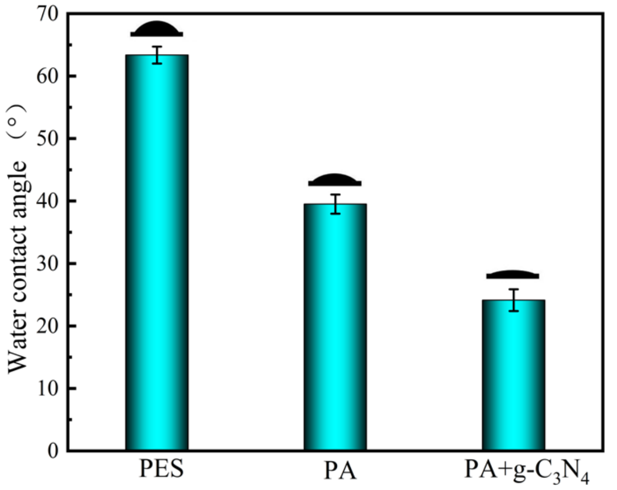

Supplementary Figure 16. Contact angle of different membranes.

3.13. XRD spectra

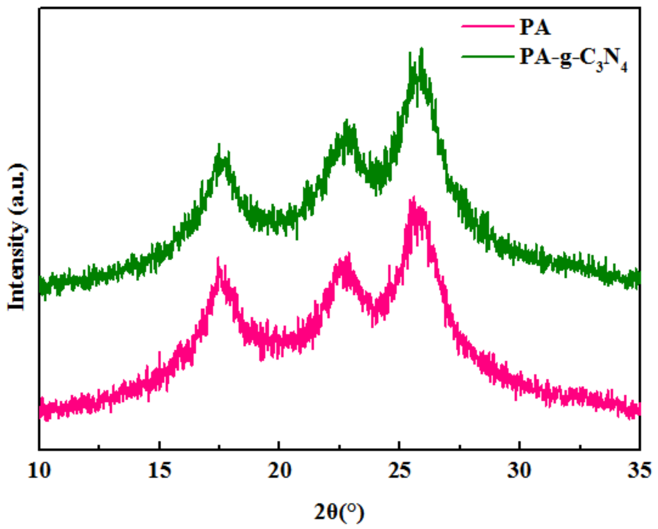

Supplementary Figure 17. XRD spectra of the membranes

4. Separation performance

4.1. Permeation and rejection of salts

As shown in Supplementary Table 6, the permeance gradually increases with the increase of TMC concentration, and reaches the maximum at 0.4 wt% under high Na_2SO_4 rejection of 99%. Then the permeance decreases again after 0.5 wt% TMC. It is known that TMC concentration plays a role in the final permeation rate of the polyamide layer. The interfacial polymerization is governed by the diffusion of monomers from both phases to the oil/water interface and the reaction rate of the interfacial polymerization[1,2]. Higher TMC concentration leads to the fast formation of a dense layer with a high density of residual carboxylic groups on the surface. Moreover, in this study, the fast reaction caused by higher TMC concentration may retard the diffusion of PIP, thus impacting the formation of nanoscale ordered structure. As is shown in Supplementary Figure S11, the nanoscale structure is most regular and substantial at high TMC concentration of 0.4 wt%, likely because the fast formation of dense layer impedes the diffusion of PIP, leading to a more ordered structure, which contributes to larger membrane area and thus higher permeation rate as is discussed in the main text.

Supplementary Table 6. The permeance of the membranes with different TMC concentrations.

Different TMC concentration (wt%)	Permeance (L $m^{-2}\cdot h^{-1}\cdot bar^{-1}$)
0.1	32 ± 1
0.2	56 ± 1
0.3	73 ± 2
0.4	103 ± 2
0.5	75± 1

Supplementary Table 7 gives the permeance comparison of the PA-g-C_3N_4 membranes with

different g-C_3N_4 concentrations. With the increase of g-C_3N_4 concentration, the permeance gradually increases, reaching the maximum at 0.135 wt% g-C_3N_4. At 0.145 wt% g-C_3N_4, the permeance decreases again, which is consistent with the less regular nanoscale structure.

It is noticed that the control membrane itself exhibited a high permeance of ~ 19 L $m^{-2}\cdot h^{-1}\cdot bar^{-1}$, while maintaining a rejection of > 99% to Na_2SO_4. This is attributed to the relatively large MWCO, high surface charge density and other structural properties of the membrane. The high density of surface charge plays an important role in rejecting Na_2SO_4 via Donnan exclusion. Similar results have been reported in existing publications by Zhang group[6]. With 114 sets of membranes, it was demonstrated that by simply tuning the compositions of PIP, TMC and surfactant in the monomer solutions, the permeance and rejection can vary in a large space. In some cases, the permeance of the membrane reached 20 L $m^{-2}\cdot h^{-1}\cdot bar^{-1}$, and the rejection to Na_2SO_4 could reach ~ 99%, which are similar to this work.

Supplementary Table 7. The permeance of the membranes with different g-C_3N_4 concentrations.

Different g-C_3N_4 concentration membranes (wt%)	Permeance (L $m^{-2}\cdot h^{-1}\cdot bar^{-1}$)
PA membrane	19 ± 1
PA-g-C_3N_4 membrane (0.075)	21 ± 1
PA-g-C_3N_4 membrane (0.115)	52 ± 2
PA-g-C_3N_4 membrane (0.125)	74 ± 2
PA-g-C_3N_4 membrane (0.135)	105 ± 2
PA-g-C_3N_4 membrane (0.145)	89 ± 1

4.2. The Cl^-/SO_4^{2-} selectivity

The separation performance of mixed salt solutions were tested by maintaining a total concentration of Cl^- and SO_4^{2-} at 2000 ppm with varying Cl^-/SO_4^{2-} weight ratio.

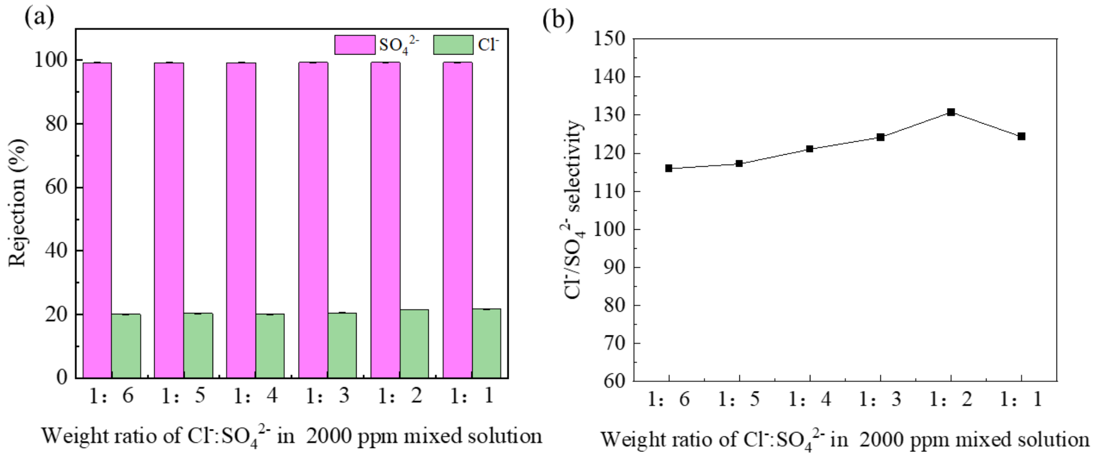

Supplementary Figure 18. Separation performance of the weight ratio of Cl^-/SO_4^{2-} in 2000 ppm mixed solution. (a) Rejection. (b) Cl^-/SO_4^{2-} selectivity.

From Supplementary Figure 18, it can be seen that the Cl^- rejection is around 20%, and the rejection to SO_4^{2-} is > 99% in all tests. The Cl^-/SO_4^{2-} selectivity can reach up to 130. The high selectivity is attributed to the negative surface charge on membranes and the suitable pore size.

4.3. Long-term experiment

As shown in Supplementary Figure 19, the long-term test of PA-g-C_3N_4 membrane showed that the permeance and rejection were stable during long operation time.

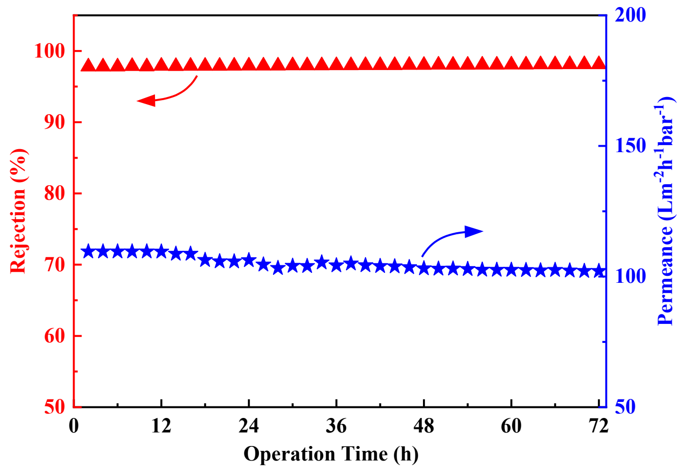

Supplementary Figure 19. Variation of the permeance and Na_2SO_4 rejection for PA-g-C_3N_4 membrane during 72 h continuous operation. (2000ppm Na_2SO_4).

4.4. Anti-fouling behaviour of PA and PA-g-C_3N_4

The anti-fouling behaviour of PA and PA-g-C_3N_4 via SEM images before and after the fouling tests with light irritation for each round were shown in Supplementary Figure 20, it can be seen that the anti-fouling behaviour of PA-g-C_3N_4 membrane was better than that of PA membrane.

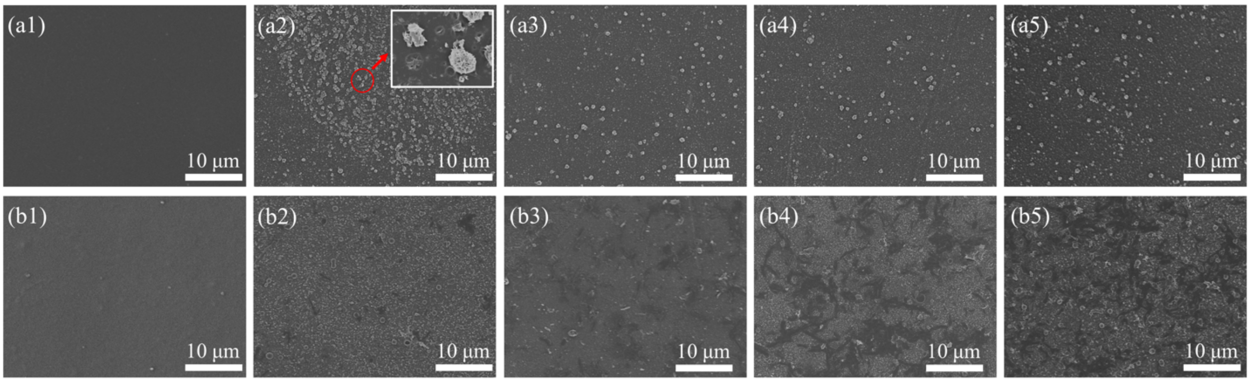

Supplementary Figure 20. SEM images of PA and PA-g-C_3N_4 before and after the fouling tests. (a1) Uncontaminated PA membrane. (a2) Contaminated PA membrane. (a3) First-round photocatalytic PA membrane. (a4) Second-round photocatalytic PA membrane. (a5) Third-round photocatalytic PA membrane. (b1) Uncontaminated g-C_3N_4 membrane. (b2) Contaminated g-C_3N_4 membrane. (b3) First-round photocatalytic g-C_3N_4 membrane. (b4) Second-round photocatalytic g-C_3N_4 membrane. (b5) Third-round photocatalytic g-C_3N_4 membrane.

Supplementary Figure 21 compares the fouled membrane surfaces after cleaning by different methods. The letters a, b and c denote to soaking, rinsing and photocatalytic cleaning respectively, and the numbers 1, 2 and 3 refer to the number of cycle. It can be seen that foulants were removed more efficiently with light irritation than the other two cleaning methods. The existence of light is crucial for membrane cleaning.

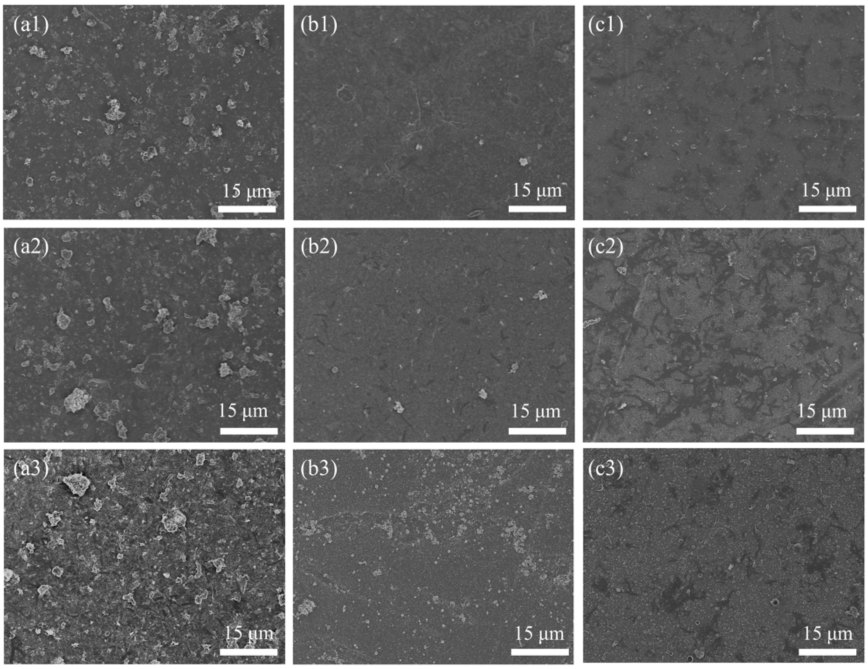

Supplementary Figure 21. The SEM images of the surface of fouled PA-g-C_3N_4 membrane after cleaning by different methods. (a1) Soak; the first cycle. (a2) Soak; the second cycle. (a3) Soak; the third cycle. (b1) Rinse; the first cycle. (b2) Rinse; the second cycle. (b3) Rinse; the third cycle. (c1) The first cycle of photocatalytic cleaning. (c2) The second cycle of photocatalytic cleaning. (c3) The third cycle of photocatalytic cleaning.

As shown in Supplementary Figure 22, the related mechanism of MB photocatalytic degradation is that electrons (e^-) and holes are generated by visible light with g-C_3N_4. The generated e^- can react with dissolved oxygen (O_2) to form superoxide radical anion O_2^-. Then h^+ and O_2^- degrade the MB to CO_2 and H_2O, membrane surface becomes clean again.

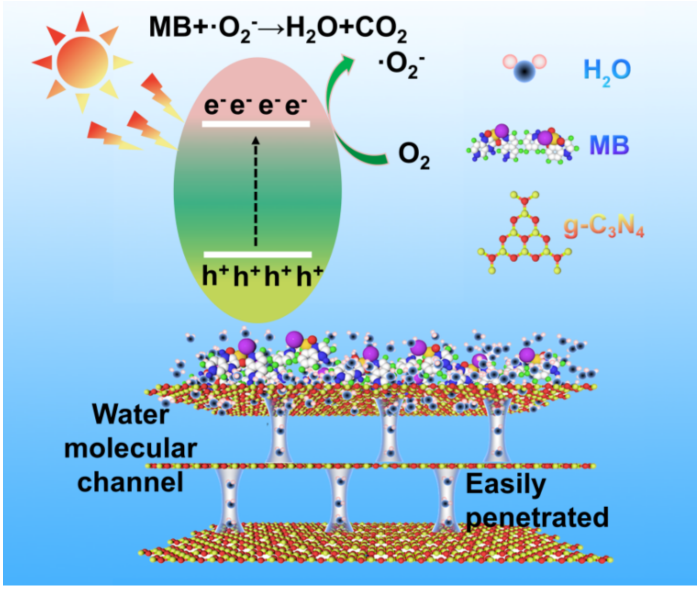

Supplementary Figure 22. The mechanism of photocatalytic degradation of methylene blue on the PA-g-C_3N_4 membrane.

As shown in Supplementary Figure 23a, the MB absorbance in the solution barely changes for the PA membrane. The absorbance decreases substantially with the PA-g-C_3N_4 membrane (Supplementary Figure 23b). Without light, no change is observed (Supplementary Figure 23c). In addition, the FTIR spectra of the PA-g-C_3N_4 membrane shows no change before and after irradiation (Supplementary Figure 23d).

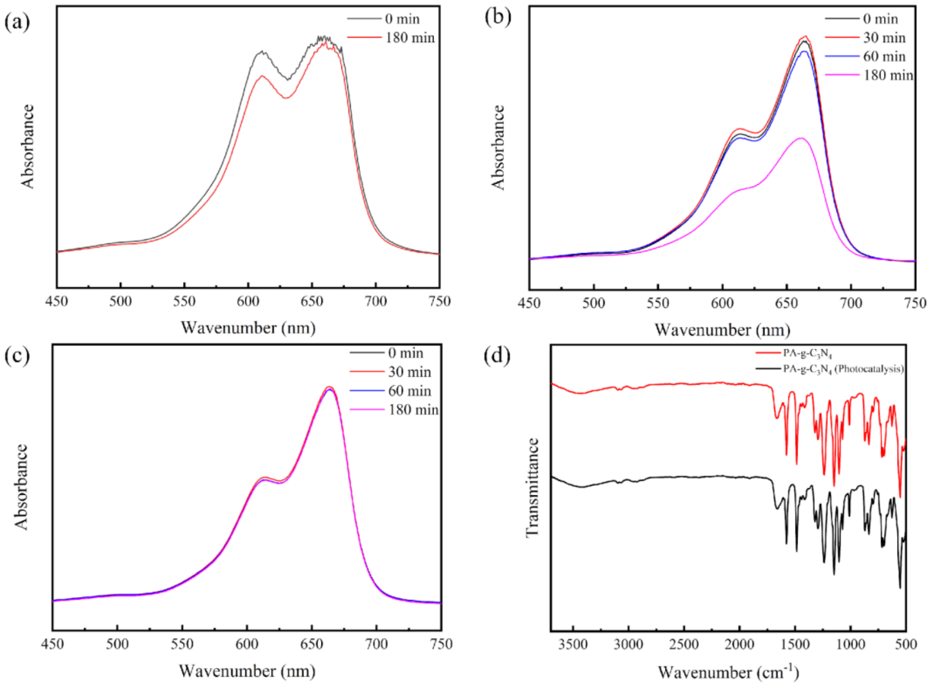

Supplementary Figure 23. The UV-Vis spectra of the membranes after immersion in the methylene blue solution and then exposed to light for different durations. (a) PA membrane with exposure to light; (b) PA-g-C_3N_4 membrane with exposure to light. (c) PA-g-C_3N_4 membrane kept in the dark. (d) The FTIR of PA-g-C_3N_4 membrane before and after exposure to light.

5. CFD results of the permeation through nanoscale ordered structure

As illustrated in Supplementary Figure 24, the introduction of hollow structures leads to the increase in observed average flux. Besides, shortening the distance between hollow structures further increase the observed average flux. It indicates that the hollow cone structure can contribute to higher total flux due to the increase in surface area.

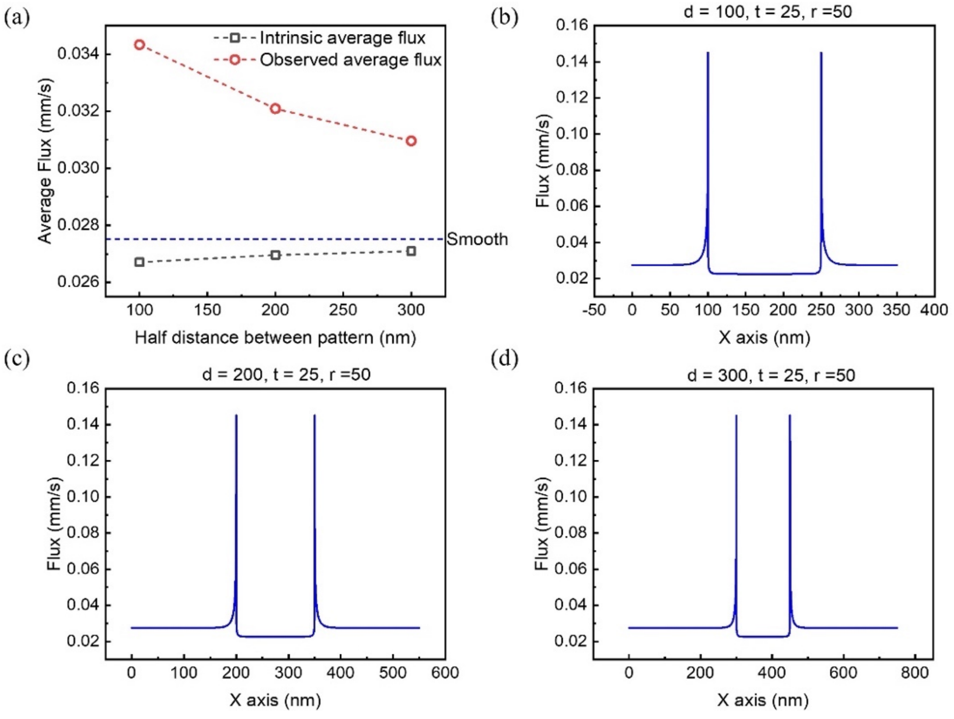

Supplementary Figure 24. (a) Average flux of smooth membrane and membranes with hollow structures. (b)-(d) Flux distribution along X axis of membranes with hollow structures. The half distances between hollow structures are (b) 100 nm, (c) 200 nm and (d) 300 nm.

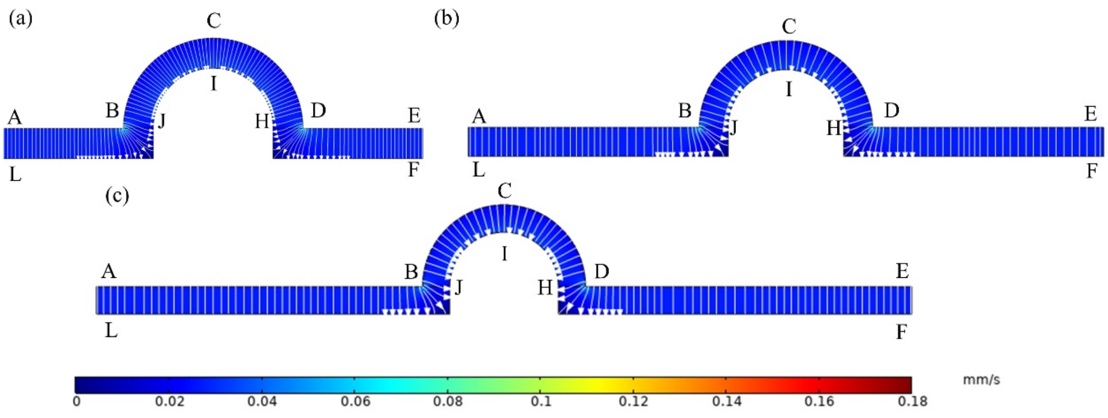

Supplementary Figure 25. Flux distribution and streamlines of membranes with hollow structures. The half distances between hollow structures are (a) 100 nm, (b) 200 nm and (c) 300 nm.

The flux distribution along the membranes shows that high local flux can be observed near the edge of hollow structures (Supplementary Figure 25, point B and D), which can be explained by the high pressure drop rate along BJ. On the contrary, hollow structure itself (BCD) shows a relatively low local flux due to the slow pressure drop rate inside the structure (Supplementary Figure 25 and 26). Overall, the high local flux near the structure edges may be outweighed by the lower flux inside the hollow structure. As a result, the intrinsic average flux decreases. Nonetheless, it is noteworthy that due to the enlarged surface area, the total (observed) flux in hollow cone membranes is higher than that in smooth membranes. Overall, the hollow cone structure contributes positively to membrane permeation.

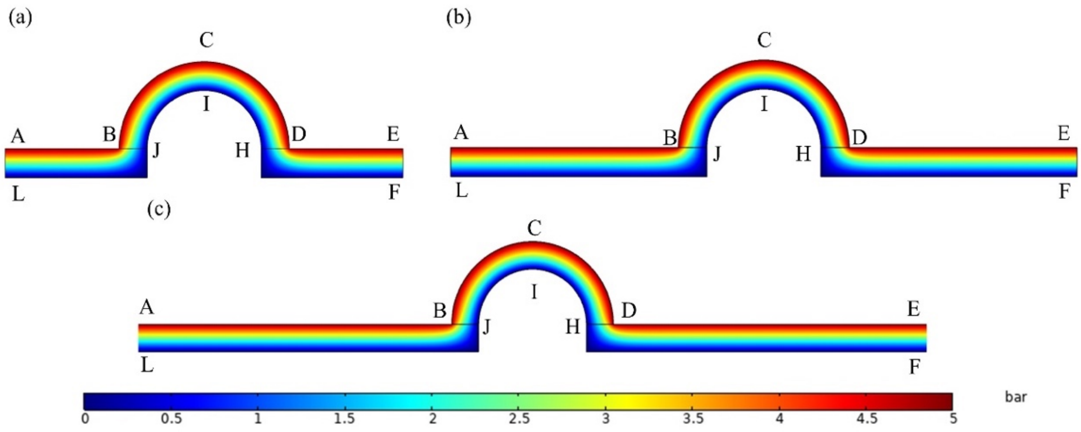

Supplementary Figure 26. Pressure distribution of membranes with hollow structures. The half distances between hollow structures are (a) 100 nm, (b) 200 nm and (c) 300 nm.

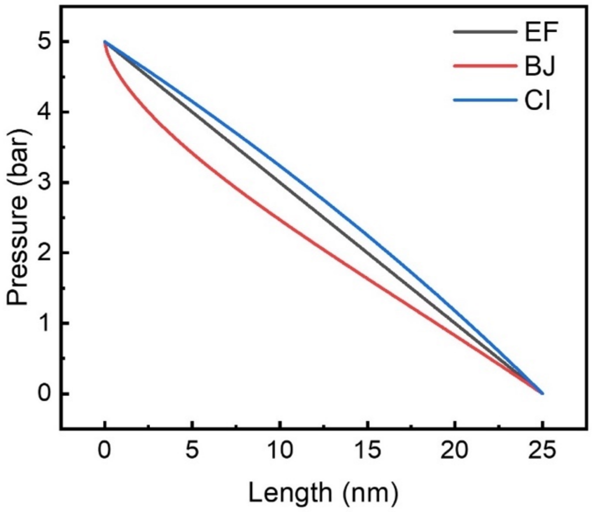

Supplementary Figure 27. Pressure drop along EF, BJ and CI.

6. Supplementary References

1. Morgan, P. W. Condensation Polymers: By Interfacial and Solution Methods (Interscience, New York, 1965.
2. Wittbecker, E. L. et al. Morgan, P. W. Interfacial polycondensation. I. *J. Polym. Sci., Polym. Phys*. Ed. **40**, 289 -297 (1959).
3. Morgan, P. W., Kwolek, S. Interfacial polycondensation. II. Fundamentals of polymer formation at liquid interfaces. *J. Polym. Sci., Polym. Phys*. Ed. **40**, 299-327 (1959).
4. Yuan, F. et al. Formation–structure–performance correlation of thin film composite membranes prepared by interfacial polymerization for gas separation. *J. Membr. Sci*. **421-422**, 327-341 (2012).
5. Yu, X. et al. Novel tertiary amino containing thin film composite membranes prepared by interfacial polymerization for CO_2 capture. *J. Membr. Sci*. **362**(1-2), 265-278 (2010).
6. Deng, H. et al., Machine learning guided polyamide membrane with exceptional solute–solute selectivity and permeance, *Environ. Sci. Technol.*, 2022, 10.1021/acs.est.2c05571.
